# The exploration of mitochondrial‐related features helps to reveal the prognosis and immunotherapy methods of colorectal cancer

**DOI:** 10.1002/cnr2.1914

**Published:** 2023-10-30

**Authors:** Yun‐hui Xie, Hui‐zhong Jiang

**Affiliations:** ^1^ Center of Gastrointestinal and Minimally Invasive Surgery, Department of General Surgery, The Third People's Hospital of Chengdu Affiliated Hospital of Southwest Jiaotong University Chengdu China; ^2^ College of Graduate Guizhou University of Traditional Chinese Medicine Guiyang China

**Keywords:** colorectal cancer, immune landscape, mitochondrial, prognosis

## Abstract

**Background:**

Cancer cell survival, proliferation, and metabolism are all intertwined with mitochondria. However, a complete description of how the features of mitochondria relate to the tumor microenvironment (TME) and immunological landscape of colorectal cancer (CRC) has yet to be made.

We performed subgroup analysis on CRC patient data obtained from the databases using non‐negative matrix factorization (NMF) clustering. Construct a prognostic model using the mitochondrial‐related gene (MRG) risk score, and then compare it to other models for accuracy. Comprehensive analyses of the risk score, in conjunction with the TME and immune landscape, were performed, and the relationship between the model and different types of cell death, radiation and chemotherapy, and drug resistance was investigated. Results from immunohistochemistry and single‐cell sequencing were utilized to verify the model genes, and a drug sensitivity analysis was conducted to evaluate possible therapeutic medicines. The pan‐cancer analysis is utilized to further investigate the role of genes in a wider range of malignancies.

**Methods and Results:**

We found that CRC patients based on MRG were divided into two groups with significant differences in survival outcomes and TME between groups. The predictive power of the risk score was further shown by building a prognostic model and testing it extensively in both internal and external cohorts. Multiple immune therapeutic responses and the expression of immunological checkpoints demonstrate that the risk score is connected to immunotherapy success. The correlation analysis of the risk score provide more ideas and guidance for prognostic models in clinical treatment.

**Conclusion:**

The TME, immune cell infiltration, and responsiveness to immunotherapy in CRC were all thoroughly evaluated on the basis of MRG features. The comparative validation of multiple queues and models combined with clinical data ensures the effectiveness and clinical practicality of MRG features. Our studies help clinicians create individualized treatment programs for individuals with cancer.

## 1 INTRODUCTION

Ten percent of all cancer fatalities each year are attributable to colorectal cancer (CRC), making it one of the most prevalent forms of the disease.^1^, ^2^ Despite the development of early screening, better referral pathways, effective primary surgery, systemic chemotherapy, and biotherapy, patients with CRC now have a better chance of surviving following diagnosis.^3^ Immune checkpoint inhibitor (ICI) therapy's widespread use is especially relevant.^4^ The yearly death rate of CRC patients is still rather high, though.^5^ Thus, it is of utmost importance to investigate and develop novel efficient biomarkers for CRC to enhance patient prognosis and the efficacy of therapy.

Mitochondria are not only the “cell power source” in eukaryotic cells but also the organelle involved in the complex process of driving cell fate.^6^ They participate in cell metabolism, differentiation, and programmed cell death through metabolic pathways.^7^, ^8^, ^9^ Mitochondria have a vital role in the maintenance and progression of illness, especially in cancer. By facilitating metabolic switch to glycolysis, mitochondria ensure cancer cells remain alive in the tumor microenvironment (TME),^10^, ^11^ enhancing redox homeostasis,^12^, ^13^ providing pH homeostasis,^14^ participating in tumor dormancy regulation mechanisms,^15^, ^16^ cancer cell autophagy,^17^ mitochondrial hijackings,^18^ and promoting angiogenesis in the TME.^19^, ^20^ At the same time, mitochondria promote tumor progression by assisting in genomic instability,^21^, ^22^, ^23^, ^24^ taking part in activities that prevent cell cycle arrest,^25^ and epithelial mesenchymal transition (EMT).^26^ Targeted anti‐mitochondrial therapy is currently one of the research hotspots. Researchers have also revealed that cancer cells' mitochondria aid in their ability to evade the immune system by creating an acidic microenvironment,^27^ glucose depletion,^28^ recruiting myeloid‐derived suppressor cells (MDSCs),^29^ contributing to the manifestation of immunological checkpoints,^30^ and taking role in the suppression of the MHC‐I expression.^16^, ^31^ And inducing the development of chemotherapy drug resistance^32^ leads to a decrease in clinical treatment benefits for cancer patients. Therefore, finding effective strategies to improve the body's immune response and therapeutic resistance can help improve the clinical treatment effectiveness of patients and prolong their survival time. The features of mitochondrial‐related genes (MRGs) have been validated as biomarkers for predicting multiple cancers prognosis and treatment.^33^, ^34^, ^35^, ^36^, ^37^ However, full investigation of MRGs' prognostic and immunological usefulness in CRC has yet to be conducted. Moreover, the link between mitochondria and CRC's TME has not yet been fully elucidated.

This research used information from the Cancer Genome Atlas (TCGA) and the Gene Expression Omnibus (GEO) databases for its analysis. Patients with CRC underwent subtype categorization using non‐negative matrix factorization (NMF) clustering, and a predictive model was built using risk scores derived from MRG features. We investigated the utility of the MRG risk score in clinical pathology, investigated the association between the MRG risk score and the TME, the immune landscape, and drug sensitivity, and finally compared the effect value of prognosis models based on the MRG risk score to those relying on other models and confirmed the expression level of model genes using immunohistochemistry. Single‐cell RNA sequencing (scRNA‐seq) was used to investigate the relationship between model genes and immune cells. Finally, the use of MRGs as biomarkers and their prognostic significance in pan‐cancer were reviewed.

## 2 MATERIALS AND METHODS

### 2.1 Data collection and identification of MRG

Using the TCGA (https://portal.gdc.cancer.gov/) and GEO (https://www.ncbi.nlm.nih.gov/geo/) databases, researchers were able to access transcriptome data, clinical data, and mutation data from CRC samples and normal tissue samples. The TCGA cohorts includes TCGA‐COAD (41 normal tissue samples, 480 tumor tissue samples) and TCGA‐READ (6 normal tissue samples, 161 tumor tissue samples). The GEO cohorts contains GSE17538 (238 samples)^38^ and GSE39582 (585 samples).^39^ In the process of organizing clinical data, samples without a survival time and samples with a survival time of 0 are excluded. The collection of MRGs are organized through the MITOMAP (https://www.mitomap.org/MITOMAP). By removing duplicate genes, we can obtain an overall of 1592 MRGs.

### 2.2 The Gene Ontology and the Kyoto Encyclopedia of Genes and Genomes enrichment analysis based on differentially expressed genes

We used the “limma” package^40^ to screen for differentially expressed genes (DEGs) based on MRG between normal and tumor tissue samples using |logFC| > 1 and FDR < 0.05 as criteria. The ggplot2 package draws volcanic maps. To further understand the potential biological functions, cellular localization, and pathways of the selected DEGs, we ran Gene Ontology (GO) and Kyoto Encyclopedia of Genes and Genomes (KEGG) functional enrichment analyses using the “clusterProfiler” R package.

### 2.3 NMF clustering, identification of molecular subtypes, and immune score analysis

We used the “survival” and “NMF” packages to extract genes linked to patient survival and prognosis by univariate Cox analysis, and then did molecular subtype clustering analysis on these genes using NMF analysis, all based on the expression of DEGs based on MRG. Clusters of molecular subtypes were limited to a value between 2 and 10. Overall survival (OS) and progression‐free survival (PFS) rates were compared among MRG clusters, and the data were plotted on a Kaplan–Meier curve for visual presentation. Using a heat map, we can see how certain MRG clusters are linked to specific clinical pathological characteristics. Calculating TME scores across MRG clusters and distinguishing between them by StromalScore, ImmuneScore, ESTIMATEScore, and TumorPurity is done using the “ESTIMATE”.^41^ To compare the levels of immune cell infiltration into the various MRG clusters, we utilized the “MCPcounter”.^42^ Display the immune response subtypes corresponding to different MRG clusters through Sankey plots.

### 2.4 Establishment of prognostic features for CRC based on MRG risk score

Create a Lasso Cox regression algorithm‐based prediction model using the “caret” and “glmnet” packages. Select relevant genes, build a prognostic model in the validation set, then compute the MRG risk score using multivariate Cox analysis. Here, coefi is the risk coefficient and EXPI is the expression level of each gene, hence the formula for calculating the risk score is risk score = ∑(EXPI * Coefi). Using the median risk score as the dividing line, the TCGA cohort sample was stratified into high‐risk and low‐risk subgroups. The prognostic model's prediction efficacy and precision were tested and verified using external validation sets consisting of survival and clinical data from various separate GEO cohorts, with the analysis of the GEO cohort performed utilizing the cutoff values from the TCGA cohort's training set. Kaplan–Meier graphs and receiver operating characteristic (ROC) curves indicated the prognostic impact of OS rate, PFS rate, and predictive model in the TCGA and GEO cohorts.

### 2.5 Subgroup hierarchical analysis of clinical pathological characteristics in predictive models

Determine how well prognosis may be predicted using predictive models for various clinical pathology variables. According to different classification criteria, different clinical pathological features were analyzed by subgroup stratification, including gender, stage, T, N, and M. And use box and bar charts to show how MRG risk scores correlate with various aspects of clinical pathology. Improve the use of prognostic models in clinical settings by comparing the clinical pathological features of low‐ and high‐risk groups of patients using heat maps.

### 2.6 Construction and validation of independent prognosis and nomogram for MRG risk score

We performed univariate and multivariate Cox regression analysis, as well as a forest plot, to see whether the MRG risk score may be employed as a standalone prognostic indicator for CRC patients. The “rms” package is used to draw predictive nomograms, which include clinical pathological features and risk scores for MRG. The calibration curve demonstrates that the 1‐, 3‐, and 5‐year OS rates are consistent with expectations, and the ROC curve demonstrates the accuracy of column charts in forecasting survival rates. To more accurately gauge nomogram’ prognostic value, the “ggDCA” package is used for decision curve analysis (DCA).

### 2.7 Compare and verify predictive performance with other models

Two studies, Qi^43^ and Du,^44^ respectively, screened 9 and 3 genes related to iron death to construct prognosis models for CRC patients. Referring to the method of constructing MRG risk score prognostic model stores, we obtained gene expression data corresponding to these two predictive models from the TCGA cohort, constructed a multifactor regression model, calculated the corresponding risk scores, and divided them into the two groups. Both the ROC curve and the Kaplan–Meier curve were used to illustrate the prognostic accuracy of the two models. Calculate the consistency index (C‐index) of these two models and our MRG risk scoring model separately, and a higher C‐index indicates a better prognostic effect. The Restricted Survival Time (RMST) curve is used to evaluate and compare the performance and differences between models.

### 2.8 Biobased enrichment analysis and immune landscape analysis

The “c2. cp. kegg. v7.4. symbols” and “c5. go. v7.4. symbols. gmt” files were set up to run GO and KEGG enrichment analysis on the two groups, respectively, and display the results of the top five enrichment rankings in order to further investigate the biological processes (BPs) and enrichment pathways of the prognosis model based on MRG risk score. We used the “GSEABase” and “GSVA” packages^45^ for GSVA analysis to supplement the results of KEGG pathway enrichment in two groups. At the same time, we comprehensively analyzed the TME and immune landscape of the MRG risk score. The TME between the two groups is realized through the “ESTIMATE” package. Visualizing the proportional difference and correlation of 22 immune‐related cell subtypes between the two groups using the CIBERSORT deconvolution algorithm^46^ and analyzing the prognostic value of immune cell infiltration level using the MRG risk score for CRC patients in combination with clinical survival time. The MCP‐counter algorithm may enhance data from other immune cell infiltration algorithms and make a connection between low‐ and high‐risk categories of immune cells via the use of bubble charts. Next, we conducted single‐sample gene set enrichment analysis (ssGSEA)^47^ to assess the differences in immune‐related functions between the two groups in order to evaluate the abundance of infiltrating immune cells and functions between the two groups. Collaboratively generate heat maps for adjuvant use based on different techniques for calculating immune cell infiltration, such as TIMER,^48^ quanTIseq,^49^ xCell,^50^ and EPIC.^51^ The expression of immunosuppressive molecules known as immunological checkpoints^52^ on immune cells allows for the control of the level of immune activation. Analyzing immune checkpoint genes in tumor tissue can help determine the effectiveness of immunotherapy. We compared the expression levels of 47 immune checkpoint genes across low‐risk and high‐risk individuals to identify potential connections and discrepancies. We gathered data from the TIDE (http://tide.dfci.harvard.edu) in order to compare the MRG risk score's ability to predict immune treatment response in low‐ and high‐risk patient groups. TCIA^47^ (https://tcia.at/home) is a database whereby one may get the findings of the TCGA cohort's immunotherapy response. The immunophenoscore (IPS) of CRC patients may be utilized to evaluate differences in immunotherapy response between the two groups.

### 2.9 Microsatellite instability, tumor cell stemness (CSC index), and tumor mutational burden were analyzed for their correlations with the risk score

Although breakthrough progress has been made in ICI therapy targeting immune checkpoints, it does not necessarily have ideal efficacy in all patients. At present, immunotherapy mainly identifies three biomarkers, namely tumor mutational burden (TMB), microsatellite instability (MSI), and PD‐L1 expression. TMB is an indirect indicator of a tumor's potential to generate new antigens and a predictor of immunotherapy's success against certain tumor types. Thus, we employed the “maftools” suite of programs to compare somatic mutation findings in two groups. Oncoplots comparing mutation rates between the two groups across the first 20 genes. The packages “ggpubr” and “ggExtra” were used to examine the association between TMB and MRG risk scores. We also looked at how low and high risk scores, the MSI, and the CSC index are related to one another.

### 2.10 Correlation and difference analysis of risk scores and genes related to cuproptosis, multiple drug resistance, TIS, radiotherapy and chemotherapy, and disulfidptosis

Mitochondria can participate in cell proliferation, metabolism, and programmed cell death through various metabolic pathways. To better predict the biological significance of the prognostic model based on the MRG risk score we created on cells, we investigated the link among the MRG risk score and gene expression associated with hot spot death modes such as cuproptosis^53^ and disulfidptosis.^54^ Radiotherapy and chemotherapy are made more challenging by tumor heterogeneity, and multiple drug resistance in tumors is the primary cause for the failure of tumor treatment.^55^ Thus, we next looked at the connection among the MRG risk score and genes involved in resistance to numerous drugs^56^ as well as radiation and chemotherapy.^57^, ^58^ Predicting the efficacy of ICIs combination treatment is possible with the use of the tumor inflammation signature (TIS), and genes associated with the TIS may act as useful biomarkers for ICIs combination therapy.^59^ Our analysis of the association among two groups and the expression of 18 TIS‐related genes may help us better understand the predictive validity of the MRG risk score in clinical therapy.

### 2.11 Drug sensitivity analysis

Semi‐inhibitory concentration (IC50) values are utilized in the “pRRophetic”^60^ to forecast the therapeutic value of chemotherapy and targeted medicines in low‐ and high‐risk patient categories.

### 2.12 Expression level and immunohistochemistry validation of model genes

From the TCGA cohort, we retrieved and compared the protein levels of prognostic model genes in both normal and malignant tissues. Integrate data from the Human Protein Atlas (HPA) database (https://www.proteinatlas.org/) to validate the protein expression of five model genes by obtaining immunohistochemistry results from tumor and normal tissues.^61^

### 2.13 Validation of model genes in single‐cell RNA sequencing

We applied the TISCH2^62^ (http://tisch.comp-genomics.org/home/) to obtain CRC‐ScRNA‐Seq data for GSE166555. This website can analyze single cell data from existing publicly available databases through the provided gene set, autonomously cluster cells, complete clustering annotations based on identified cell markers and alignment results, and present the distribution results of target genes in cells.

### 2.14 Pan‐cancer analysis of model genes

This research aims to explore the potential of the MRG risk score as a pan‐cancer prediction model gene. Using heat maps, we showed how model genes are expressed in different malignancies and how their expression is correlated using analysis of five genes. Finally, forest plots were used to display the results of a Cox analysis into the prognostic risk of model genes in pan‐cancer.

### 2.15 Statistical analysis

The analysis based on the R package in this study was completed using R version 4.2.3. The log rank test was used to compare OS and PFS rates between the TCGA and GEO cohorts. Correlations were analyzed using the Pearson test. Two groups of data may be compared using the Wilcoxon test. *p* < .05 is considered statistically significant.

## 3 RESULTS

### 3.1 Identification and NMF cluster typing of MRGs

**FIGURE 1:**
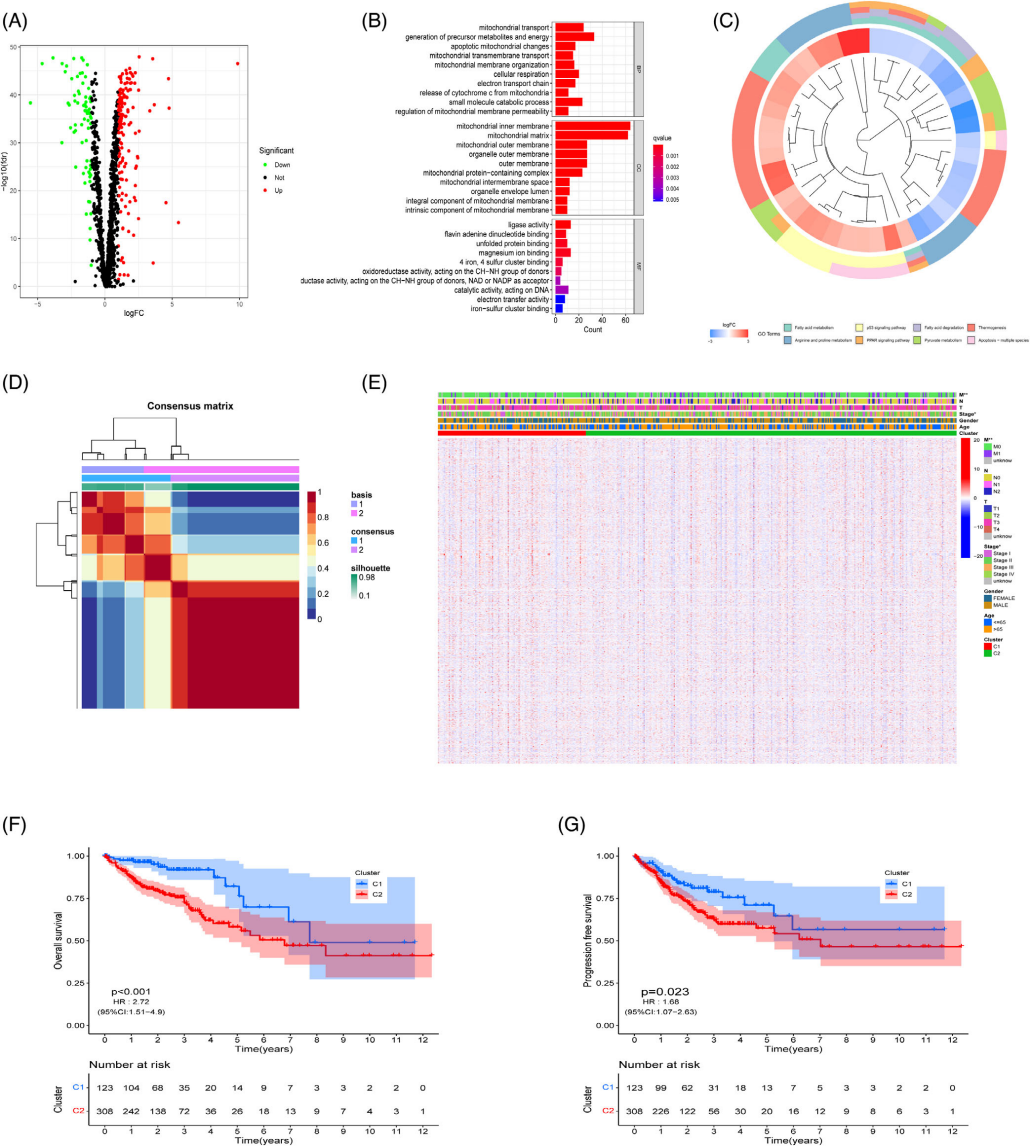
Differentially expressed genes (DEGs) extraction and identification of mitochondrial‐related genes (MRGs). (A) Volcano plot of the DEGs of MRGs; (B) Gene Ontology enrichment analysis; (C) Kyoto Encyclopedia of Genes and Genomes analysis of the related pathways; (D) Heatmap of the NMF clustering (*k* = 2); (E) Heatmap of differences in clinicopathological factors between the two different Clusters; (F, G) The Kaplan–Meier curve of overall survival and progression‐free survival rates among the Clusters. **p < .01; *p < .05.

A total of 226 mitochondrial‐related differential genes, comprising 151 upregulated genes and 75 downregulated genes, were found using differential analysis of 1592 MRGs in the TCGA cohort, and the findings were shown through a volcano map (Figure 1A). The BPs and pathways associated with MRGs may be elucidated by doing GO and KEGG enrichment analysis on MRG‐DEGs. The analysis of GO discovered that in the BP, it involves mostly the generation of precursor metals and energy and mitochondrial transporters (Figure 1B); in the cellular component (CC), it mainly involves mitochondrial inner members and matrix; and in the molecular function (MF), it mainly involves ligase activity. The results of KEGG are shown in the cluster diagram (Figure 1C), which shows that MRGs are mainly enriched in fatty acid metabolism and degradation, arginine and profile metabolism, pyruvate metabolism, thermogenesis, apoptosis, the p53, and the PPAR signaling pathway. The majority of these enriched pathways are concerned with metabolic and apoptotic processes. Further analysis of these DEGs identified a total of 204 prognostic‐related MRGs through univariate Cox regression combined with clinical survival data. The NMF algorithm resulted in *K* = 2 was the best choice for MRG clustering and divided these genes into two clusters, Cluster 1 (*n* = 123) and Cluster 2 (*n* = 308) (Figure 1D). In light of clinical data, we compared the differences in clinical pathological features between two MRG clusters. The heat map shows significant differences in M‐type and stage between the two clusters (*p* < .01; *p* < .05; Figure 1E). The Kaplan–Meier curve, which uses a combination of survival data, demonstrated that Cluster1 had considerably superior OS and PFS than Cluster2 among CRC patients (*p* < .001, HR: 2.72 [95% CI: 1.51–4.9]; *p* = .023, HR: 1.68 [95% CI: 1.07–2.63]; Figure 1F,G). This indicates that CRC patients in the Cluster1 cluster have better prognostic outcomes.

### 3.2 MRG based TME and immune assessment between two clusters

**FIGURE 2:**
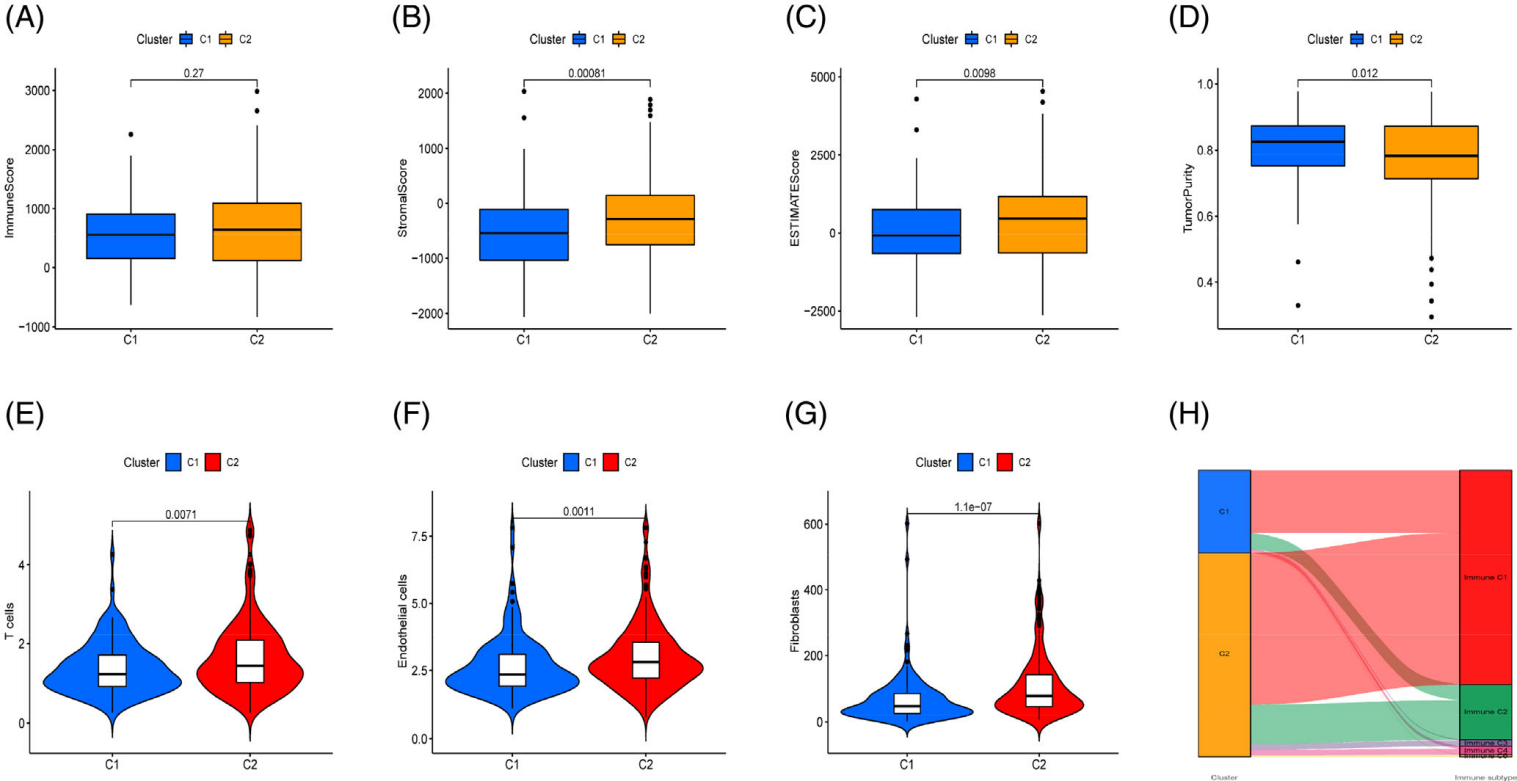
Immune‐microenvironment analysis between the cluster subtypes. (A) The ImmuneScore results between the cluster subtypes; (B) The StromalScore results between the cluster subtypes; (C) The ESTIMATEScore results between the cluster subtypes; (D) The TumorPurity results between the cluster subtypes; (E–G) Analysis of the T cells, Endothelial cells, Fibroblasts infiltration among the cluster subtypes; (H) Sankey plot of the correlation between the cluster subtypes and immune subtypes.

The purpose of this study is to determine what factors distinguish Cluster 1 from Cluster 2 in terms of survival and prognosis, we compared the TME between the two clusters and evaluated the immune invasion. TME findings showed no statistically significant variation in immunological ratings across groups (*p* = .27, Figure 2A), but Cluster 2 was higher than Cluster 1 in matrix scores and estimate scores (*p* = .00081; *p* = .0098; Figure 2B,C). However, Cluster 1 has a higher tumor purity than Cluster 2 (*p* = .012, Figure 2D). The MCP counter algorithm shows that Cluster 2 mainly exhibits higher levels of infiltration on three types of immune cells: T cells, endothelial cells, and fibroblasts (Figure 2E–G). Existing studies have identified six immune subtypes, namely immune C1–C6, through immunogenomic analysis of TCGA pancreatic cancer samples.^63^ In our study, the analysis of the immune subtypes contained in Cluster 1 and Cluster 2 showed that there was no partial aggregation of immune subtypes with immune rest (C5) in both clusters. Wound healing (C1) accounts for a large proportion in Cluster 1 and Cluster 2, while other subtypes, including IFN‐γ(C2), inflammation (C3), lymphocyte depletion (C4), and TGF‐ dominant (C6), aggregate less in both clusters.

### 3.3 Construction of a prognostic model based on MRG

**FIGURE 3:**
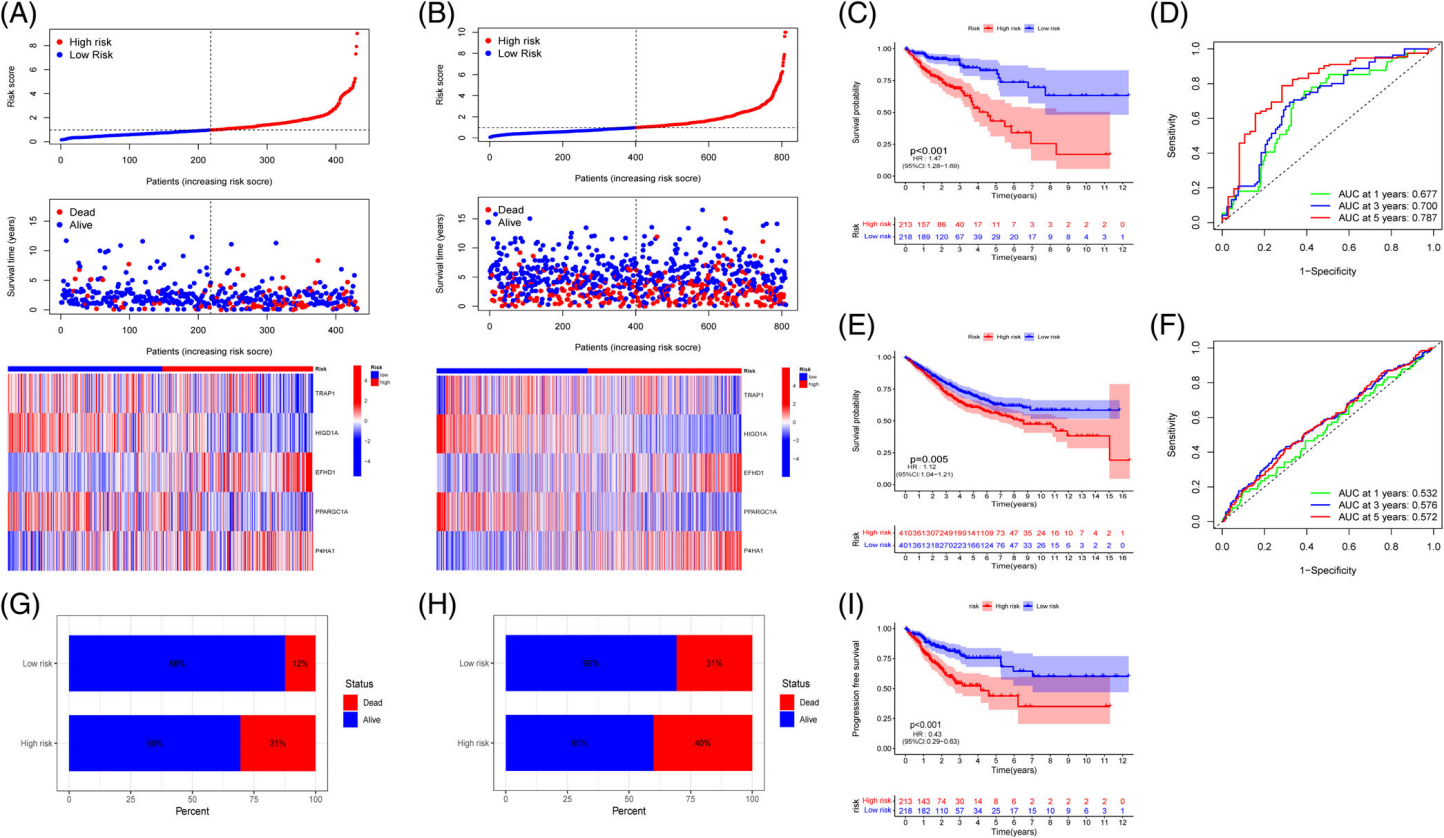
Construction and validation of the mitochondrial‐related gene risk scores. (A, C, D, G, I) Risk distribution, survival status, related gene expression, overall survival (OS) rates, receiver operating characteristic (ROC) curves, and progression‐free survival rates between the two groups in TCGA cohort; (B, E, F, H) Risk distribution, survival status, related gene expression, OS rates, ROC curves of patients between the two groups in Gene Expression Omnibus cohort.

The TCGA whole cohort's CRC samples were randomly split into a training set (*n* = 303) and a testing set (*n* = 128) with a 7:3 ratio, and there were no discernible differences in clinical pathological features between the two sets (Table S1). Using Lasso‐Cox regression analysis, we developed a model for predicting MRG outcomes (Figure S1A), and five model genes (TRAP1, HIGD1A, PPARGC1A, EFHD1, and P4HA1) were identified (Figure S1B). Among them, TRAP1, HIGD1A, and PPARGC1A are protective factors, while EFHD1 and P4HA1 are risk factors. Calculate the risk score based on MRG features through multivariate Cox regression analysis. Risk score = (−0.6794 * expression of TRAP1) + (−0.8594 * expression of HIGD1A) + (−0.4220 * expression of PPARGC1A) + (0.4966 * expression of EFHD1) + (0.7134 * expression of P4HA1). Low‐ and high‐risk segments of the TCGA cohort are defined by the median risk score as the dividing line. The internal validation (training set and test set of the TCGA cohort) and external validation (GEO cohort) are also divided into high and low risk groups using the same method, and the following analysis is performed to further evaluate the robustness of the prognosis model based on the MRG risk score. Survival rates and mortality rates for CRC patients both rise with MRG risk score, as seen by the TCGA whole cohort's risk distribution map. Meanwhile, the expression of TRAP1, HIGD1A, and PPARGC1A genes was positively connected to MRG risk score, while EFHD1 and P4HA1 were negatively correlated with MRG risk score (Figure 3A). GEO cohort (Figure 3B), training set (Figure S1C), and test set (Figure S1D) validation of the association between MRG risk score and survival status and model gene expression. Kaplan–Meier analysis of the TCGA population showed that the low‐risk group had a considerably higher OS rate than the high‐risk group (*p* < .001, HR: 1.47 [95% CI: 1.28–1.69], Figure 3C), and that the low‐risk group had a lower percentage of fatalities among CRC patients than the high‐risk group (12% vs. 31%, Figure 3G). Prognostic value was strong in the TCGA cohort, as shown by the ROC curve, with AUC values of 0.677 at 1 year, 0.700 at 3 years, and 0.787 at 5 years (Figure 3D). The prognostic worth also rises with the amount of time between the forecast and its execution. These MRG risk scores were shown to have prognostic significance, and this was further confirmed in the validation sets. Patients classified as low risk had an excellent OS rate, as seen by the Kaplan–Meier curve both in the training and test sets (*p* < .001, HR: 1.66 [95%CI: 1.42–1.95]; *p* = .003, HR: 1.12 [95%CI: 0.82–1.53]; Figure S2A,E). CRC patients at high risk of complications had a greater mortality rate than those at low risk (28% vs. 11%, 37% vs. 15%; Figure S2D,H). AUC values of 0.733, 0.714, 0.805, and 0.584, 0.670, and 0.754 at 1, 3, and 5 years were also seen in the ROC curves of the training and testing sets, indicating strong prognostic effects (Figure S2C,G). At the same time, in the GEO external cohort, The low‐risk group had a far greater OS rate than the high‐risk group (*p* = .005, HR: 1.12 [95% CI: 1.04–1.21], Figure 3E). Moreover, the high‐risk group had a greater mortality rate than the low‐risk group did among those with CRC (40%, 31%, Figure 3H). 1‐, 3‐, and 5‐year AUC values were 0.532, 0.576, and 0.572, respectively (Figure 3F). Considering the unique nature of tumor disease development, where tumor growth is to some extent the cause of tumor‐related deaths, we conducted a disease‐free progression analysis on patients in the TCGA cohort. PFS in the low‐risk group was considerably greater than in the high‐risk group, as shown by the Kaplan–Meier curve in the TCGA whole cohort (*p* < .001, HR: 0.43 [95% CI: 0.29–0.63], Figure 3I), which was confirmed in the training and testing sets (*p* = .002, HR: 0.5 [95% CI: 0.32–0.79]; *p* < .001, HR: 0.31 [95% CI: 0.15–0.65]; Figure S2B,F). The disease‐free progression period reflects the growth of tumors and can better predict and reflect clinical benefits. These findings demonstrate that the MRG risk score‐based prediction model performs well in terms of both robustness and prognostic capacity.

### 3.4 The clinical value of MRG risk score prognostic model

**FIGURE 4:**
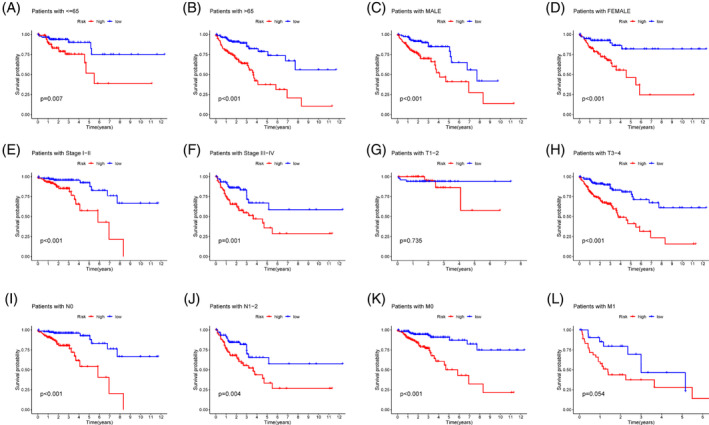
Prognosis stratified by mitochondrial‐related gene risk scores with clinicopathologic factors in colorectal cancer. (A—L) Subgroup analysis of the clinical data.

To examine the clinical prognostic efficiency of the prognostic model, we conducted stratified subgroup research on several clinical and pathological characteristics of CRC patients. There is no statistically significant difference in OS among the T1‐2 and M1 subgroups, as shown by the Kaplan–Meier curve for subgroup classification based on clinical pathological features (Figure 4G,L). Low‐risk patients had a considerably greater OS rate than those in the high‐risk group across all categories (Figure 4A–F,H–K). This demonstrates the model's strong prognostic abilities across a wide variety of clinical subgroups.

**FIGURE 5:**
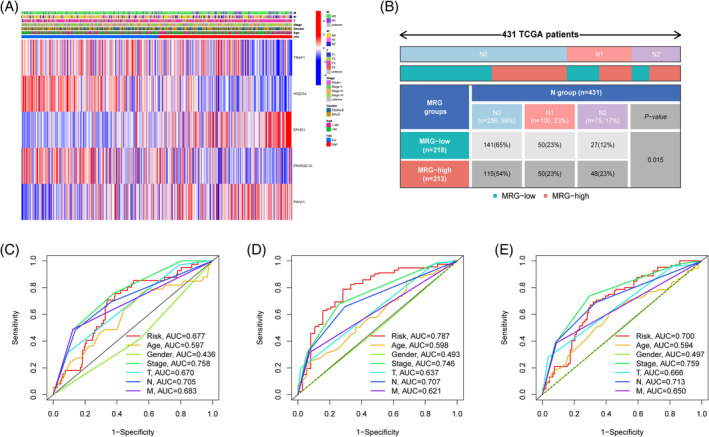
Identification of the clinical application value. (A) Clinical stratification comparison between the two groups; (B) Specific differences in the two groups in the N stage; (C–E) Risk scores predicted 1‐, 3‐, 5‐years for patients stratified by clinicopathologic factors. *p < .05.

We studied the relationship between risk scoring and clinical pathological characteristics to learn more about the clinical utility of risk scoring. A higher proportion of patients in the high‐risk CRC group had advanced stages of various clinical pathological features, while being of similar age and gender to those in the low‐risk group (Figure S3A). Subgroups with distinct clinical pathological traits are compared using risk ratings (Figure S3B), the results showed that regardless of age or gender, there were no discernible changes in risk ratings (*p* > .05). risk scores varied significantly by stage, T, N, and M (*p* < .05), demonstrating a strong link between risk scores and certain clinical pathological aspects. In response to this result, we analyzed the overall differences in clinical pathological features. Surprisingly, we discovered that the sole distinguishing factor between the two groups was T stage (*p* = .015, Figure 5A,B). This may mean that our prognostic model has greater application value in subgroup stratification of clinical pathological features, which is beneficial for analyzing the impact of MRG risk scores on specific clinical subgroup patients. Clinical pathological features within 1‐, 3‐, and 5‐years indicate high predictive performance for the MRG risk score‐based prognosis model, as shown by the ROC curve (Figure 5C–E).

### 3.5 Validation of nomograms and independent risk score prognostication

**FIGURE 6:**
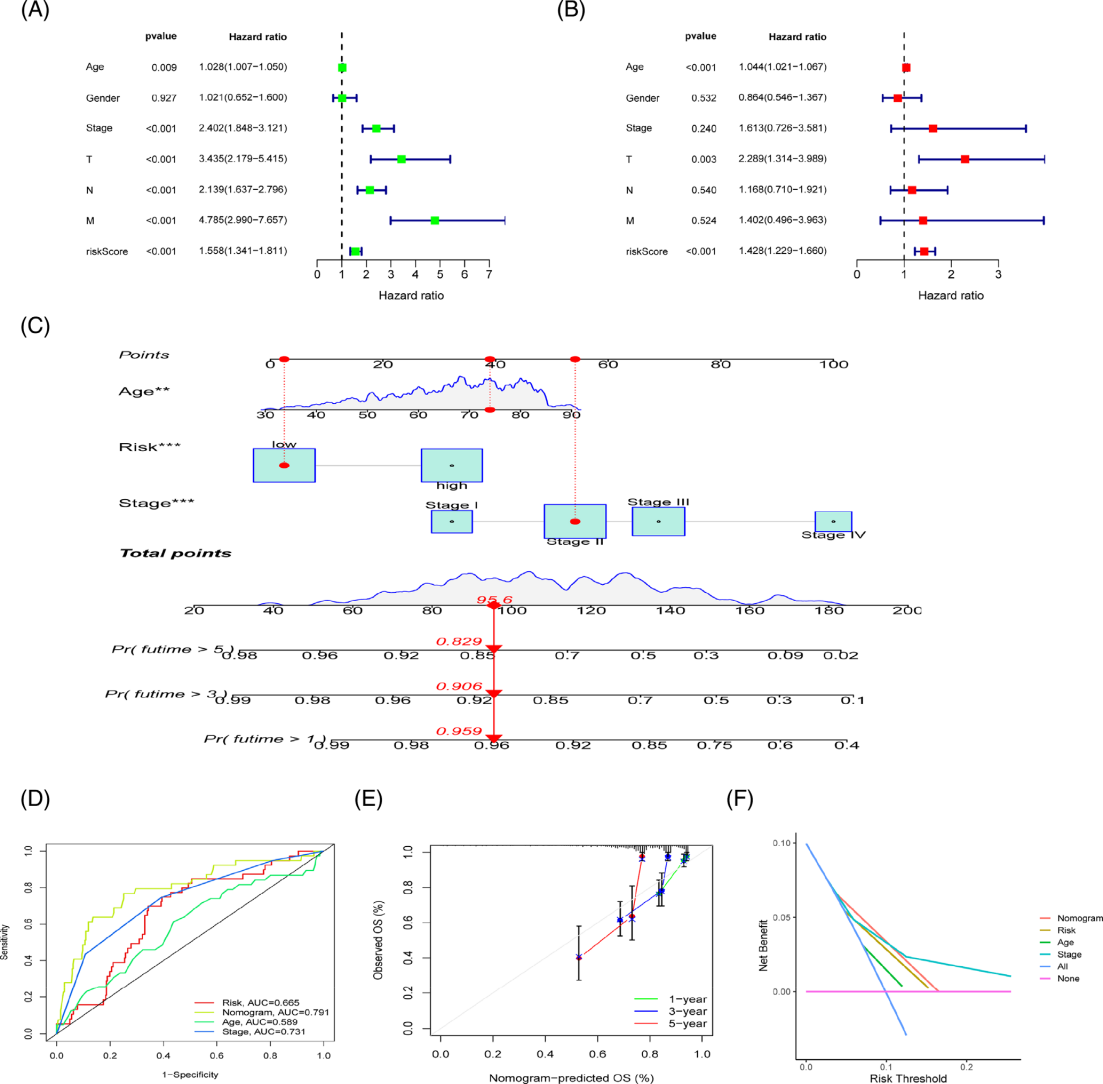
Verification of the risk scores' predictive ability. (A, B) Validation of the prognostic effect of the risk scores; (C) Survival nomograms for the TCGA cohort, estimating OS rates; (D) Predictions of the nomogram's impact using its associated ROC curves; (E) The Calibration curve of the nomogram; (F)The DCA curves used to test the prediction accuracy of the nomogram. ***p < 0.001; **p < .01.

We used univariate and multivariate Cox regression analysis to evaluate the prognostic relevance of MRG risk scores and clinical pathological features as independent factors. Excluding gender from the analysis, univariate Cox regression demonstrated that other clinical pathological characteristics and MRG risk scores may independently alter the model's predictive effectiveness (*p* < .05, Figure 6A). The predictive value of age, T, and risk score was further validated by the multivariate Cox regression findings (*p* < .05, Figure 6B). In other words, the MRG risk score may be used apart from other factors to predict how well a patient with CRC would do. Concurrently, we developed a nomogram to assess an individual's likelihood of survival (Figure 6C). The ROC curve also demonstrated the nomogram's high degree of accuracy in making predictions (AUC = 0.791, Figure 6D). Similarities between expected and actual survival are shown on the nomogram's calibration curve (Figure 6E), while the DCA curve confirms the accuracy of the nomogram (Figure 6F).

### 3.6 Comparison of predictive performance between models

**FIGURE 7:**
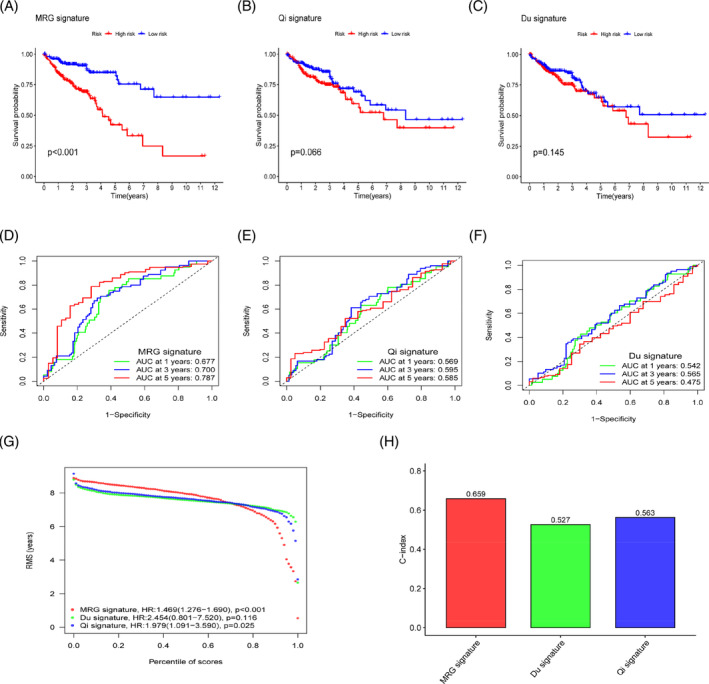
The prognostic properties of mitochondrial‐related gene (MRG) risk scores compared to other prognostic models. (A–C) Kaplan–Meier curves established by Qi, Du, and MRG; (D–F) The receiver operating characteristic curves established by Qi, Du, and MRG; (G) The Restricted Survival Time curves compared the prognostic effects of the three prognostic models; (H) The C‐index comparison of the three prognostic models.

The MRG risk score was used to create a prognosis model for patients with CRC, and this model was compared to two additional models. The Kaplan–Meier curve demonstrates that our model is superior to those developed by Qi and Du in terms of its ability to differentiate prognosis (*p* < 0.001; *p* = .066; *p* = .145; Figure 7A–C). Comparisons were made between the three models' AUCs after 1‐, 3‐, and 5‐ years. Findings demonstrate our MRG risk scoring model's improved predictive performance (Figure 7D–F). RMST is a robust and clinically interpretable summary method for the distribution of survival time. The three sets of RMST curves and C‐index results (Figure 7G,H) show that the prognostic characteristics of MRG have consistently superior performance and better clinical practicality.

### 3.7 Bioenrichment analysis based on MRG risk score

**FIGURE 8:**
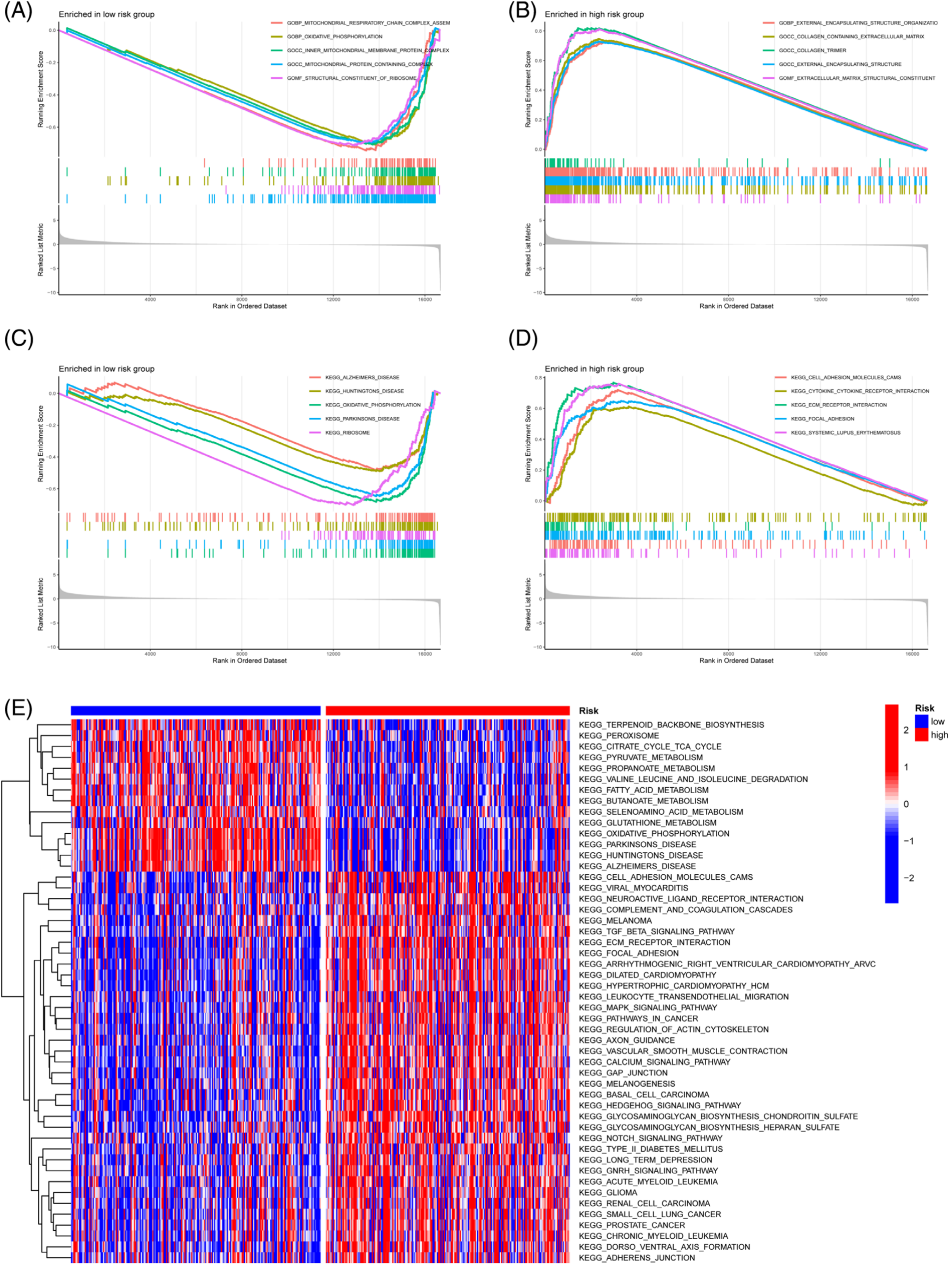
Biological enrichment results based on prognosis model genes for mitochondrial‐related gene risk factors. (A, B) Gene Ontology enrichment results for the two groups; (C, D) Kyoto Encyclopedia of Genes and Genomes (KEGG) pathway enrichment results; (E) GSVA supplementation of KEGG pathway enrichment results.

The top five BPs and pathways were identified by an examination of GO and KEGG enrichment in two groups. Low‐risk individuals had a higher propensity to be engaged in mitochondrial respiratory chain complex assembly and oxidative physiology, according to the findings of the GO enrichment analysis comparing two groups (Figure 8A), whereas matrix was the biggest contributor to the high‐risk group (Figure 8B). Low‐risk individuals were mostly enriched in KEGG illness pathways associated with the nervous system (Figure 8C), while the high‐risk group mainly focused on cell adhesion molecules (CAMs), ECM receptor interaction, and cyclokine‐cyclokine receptor interaction (Figure 8D). Our supplemental GSVA analysis of KEGG enrichment revealed that the low‐risk group was mostly engaged in metabolic‐related pathways, whereas the high‐risk group was primarily involved in a wide variety of cancer‐related pathways, typical carcinogenic‐related pathways, etc (Figure 8E).

### 3.8 Comprehensive immune landscape analysis of prognostic characteristics of MRG risk score

**FIGURE 9:**
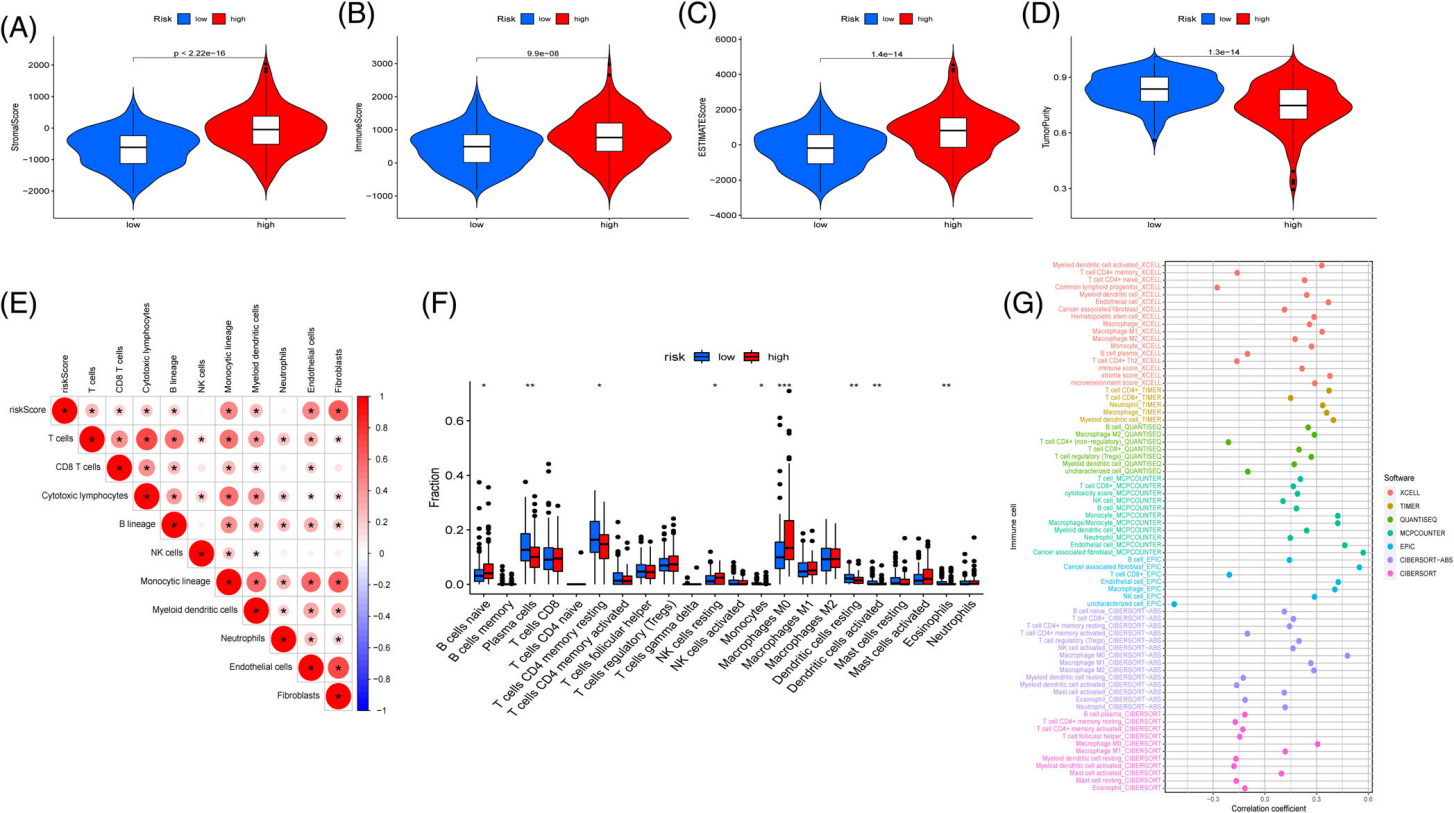
Tumor microenvironment (TME) and immune cell infiltration predicted by the prognostic model. (A–D) StromalScore, immuneScore, ESTIMATEScore, and TumorPurity results for the TME; (E) Infiltration of immune cells and their relationship to the mitochondrial‐related gene risk score; (F) Differences in immune cell levels; (G) With the use of several algorithms, the heatmap displays the variation in immune cell expression. ***p < .001; **p < .01; *p < .05.

Immunotherapy is one of the most effective means of cancer treatment. The TME can directly or indirectly affect the effects of immunotherapy. We compared the TME disparity among two groups using ESTIMATE. The high‐risk group had a higher matrix score, immunological score, and estimate score than the low‐risk group (*p* < .001, Figure 9A–C). The low‐risk group had far higher tumor purity than the high‐risk group (*p* < .001, Figure 9D). Using the MCPcounter method, we found a positive association between the MRG risk score and many subsets of immune cells: T cells, CD8 T cells, cytotoxic lymphocytes, B lineage, monocytic lineage, myeloid dendritic cells, endothelial cells, and fibroblasts (*p* < .05, Figure 9E). The CIBERSORT technique was used as a supplementary tool to compare immune cell infiltration between two groups. A negative correlation was found between the risk score and activated dendritic cells, resting dendritic cells, eosinophils, and plasma cells (*p* < .05, Figure S4A,B,D,E,F), whereas resting M0 macrophages and NK cells exhibited a substantial positive connection with the risk score (*p* < .05, Figure S4C,G). More immune cell correlation calculation methods are presented in the form of bubble charts to improve our results (Figure 9G). Plasma cells, T CD4 memory resting cells, monocytes, dendritic cells resting cells, dendritic cells activated cells, and eosinophils were more numerous in the low‐risk group, while B naive cells, NK resting cells, and M0 macrophage were more numerous in the high‐risk group (Figure 9F). We also performed a combined study of immune cell infiltration and survival time to better understand the connection among the two variables and the prognosis in two groups. Patients with a lower MRG risk score and a lower infiltration abundance of naive B cells and M0 macrophages had a better prognosis, as shown by the Kaplan–Meier survival curve (*p* = .006; *p* = .038, Figure S4H,K). Patients have a better chance of survival because to the large number of infiltrating T cells, as well as activated follicular helper and mast cells (*p* = .044; *p* = .027, Figure S4I,J).

**FIGURE 10:**
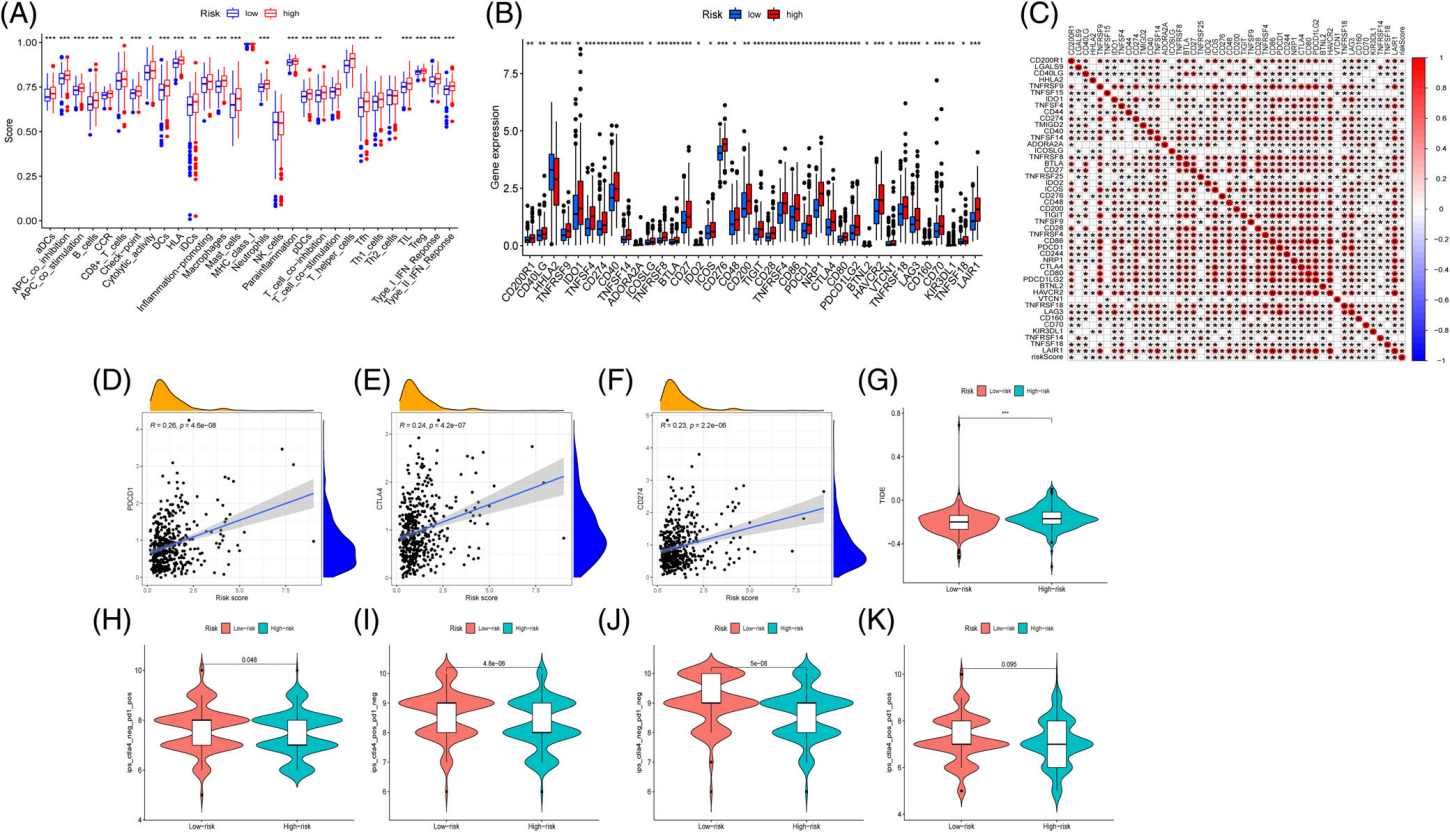
Forecasting the immune response using the mitochondrial‐related gene (MRG) risk score. (A) Analysis of immune‐related functional differences; (B) Immune checkpoint genes were differentially expressed; (C) Analysis of the connection between the MRG risk score and the immune checkpoint genes; (D– F) Key immunological checkpoint genes: correlation analysis results; (G) The TCIA analysis of the immunotherapy response results in the two groups; (H–K) The IPS analysis of the response. ***p < .001; **p < .01; *p < .05.

Out of 29 immune‐related functions, only MHC class I, NK cells, and Th2 cells showed no difference between the two groups in the ssGSEA analysis. In all other areas, however, the high‐risk group had superior immune function ratings than the low‐risk group (*p* < .05, Figure 10A). The expression levels of 38 immune checkpoint genes were found to vary between the two groups after analyzing the correlation among these genes and the MRG risk scores (*p* < .05, Figure 10B). Only one of these immune checkpoint genes, HHLA2, showed increased expression in the low‐risk group compared to the high‐risk group, whereas the expression levels of the other 37 genes were significantly greater in the high‐risk group. Forty immune checkpoint genes had a strong correlation with the MRG risk score (Figure 10C). We looked examined how CD274, CTLA4, and PDCD1 (the most common immune checkpoint genes) were associated with the risk ratings. As the risk score increased, there was a statistically significant increase in the expression of these three genes (*p* < .05, Figure 10D–F). Predicting the therapeutic efficacy of ICI (anti‐PD‐1/anti‐CTLA4) may be done using the TIDE score, which measures tumor immune dysfunction and rejection. In contrast to the low‐risk group, the high‐risk group had a higher TIDE score (*p* < .01, Figure 10G). Combining the TCIA‐obtained IPS with the MRG risk score for analysis helps clarify the favorable effects of immunotherapy on high‐ and low‐risk populations. A study looking at the usage of anti‐PD‐1 and CTLA4 found that the low‐risk group's IPS scores were significantly higher than the high‐risk group's under treatment with PD‐1 or CTLA4 inhibitors alone (*p* < 0.05; *p* < .001, Figure 10H,I). Simultaneously, the low‐risk group had a much better IPS score than the high‐risk group, and this was the case even in the absence of PD‐1 and CTLA4 inhibitor therapy (*p* < .001, Figure 10K). When PD‐1 and CTLA4 inhibitors were used combined, there was no discernible difference in IPS scores among the two groups (Figure 10J). Patient outcomes with anti‐PD‐1/anti‐CTLA4 immunotherapy were better in the low‐risk group than in the high‐risk group, as illustrated above.

### 3.9 Correlation analysis of prediction models with MSI, CSC index, and TMB

**FIGURE 11:**
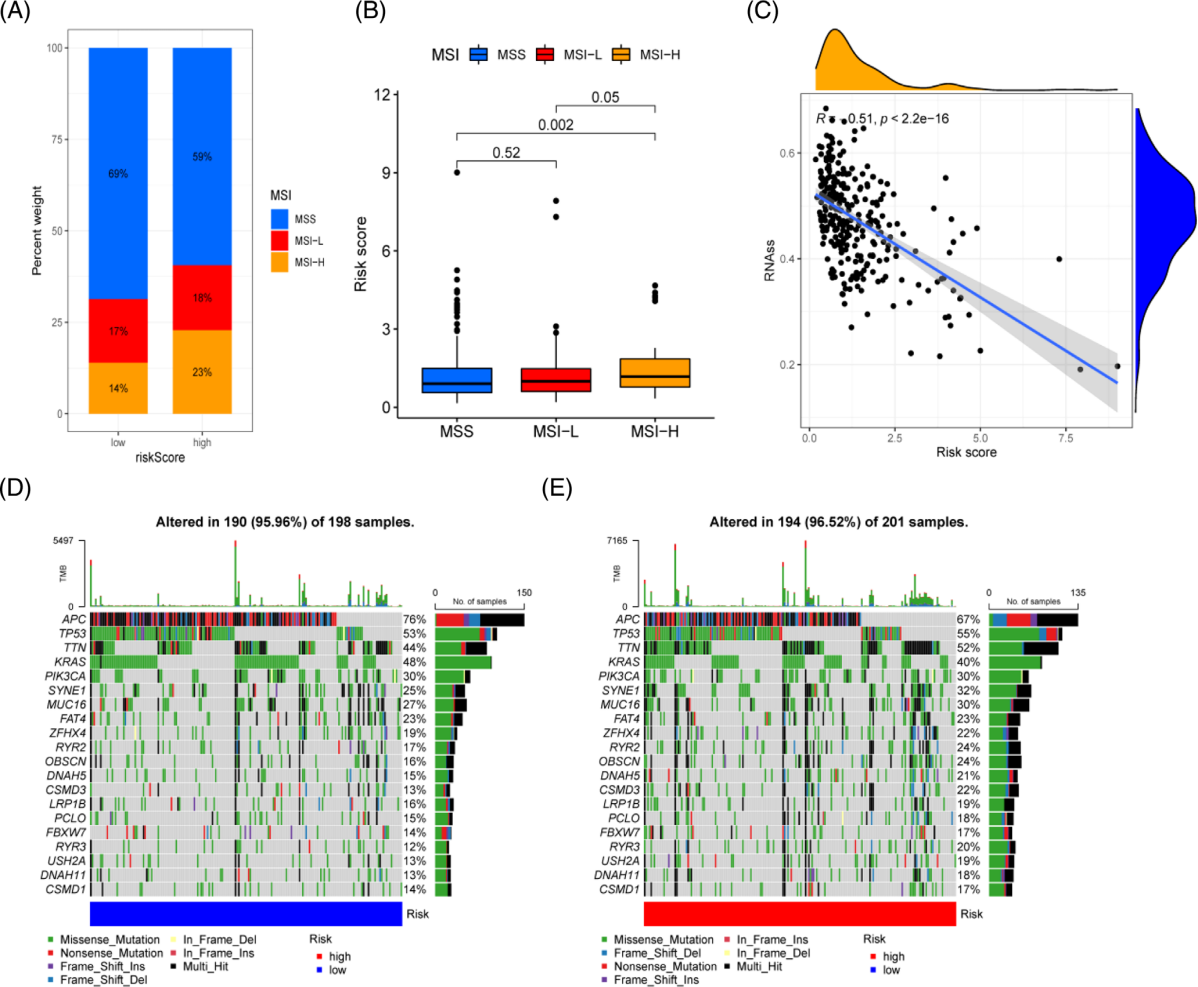
Association between mitochondrial‐related gene (MRG) risk score and microsatellite instability (MSI), tumor mutational burden, and cancer stem cells in colorectal cancer. (A, B) Connecting the MRG risk score to the MSI; (C) Correlation of MRG risk score and cancer stem cells; (D, E) Somatic mutation analysis via the MRG risk score on oncoplots.

MSI and TMB have been established in prior research to be reliable indicators for estimating immunotherapy success. The CSC index can also affect the effectiveness of patients receiving immunotherapy. Predictive value of immunotherapy in CRC patients may be improved with the use of correlation analysis among the MRG risk score and MSI, TMB, and CSC index. Patients with MSI‐H types were more prevalent in the high‐risk group than in the low‐risk group, according to this research (23% vs. 14%, Figure 11A), and the risk score of MSI‐H type patients was significantly higher than that of MSS type patients (*p* < .01, Figure 11B). The tumor cell stemness index based on RNA expression was significantly negatively in close relation to the MRG risk score (*p* < .001, Figure 11C). The oncoplot displays the results of the top 20 most common gene mutations in the high‐risk and low‐risk group samples (Figure 11D,E). The findings demonstrate that whereas mutation rates are greater across the board for the high‐risk group, they are higher for the low‐risk group in just two genes (APC and KRAS). Conversely, there was no discernible variation in TMB between the high‐ and low‐risk groups (*p* = .098, Figure S5A). On the other hand, there was a favorable connection between the MRG risk score and TMB (*p* = .013, Figure S5B). We used the MRG risk score predictive model for CRC patients and conducted a subgroup analysis combining TMB level and risk score to investigate the impact of TMB level on survival time. The data revealed that the 5‐year survival rate for CRC patients was greatest in the high mutation and low risk categories (*p* < .0001, Figure S5C). Mutation analysis of five genes included in the prognostic model revealed that missense mutations accounted for the vast majority of changes, and that the frequency of mutations in CRC samples was low (Figure S5D).

### 3.10 Correlation of the prediction model with the TIS‐, multidrug resistance‐, chemoradiotherapy‐, cuproptosis‐, and disulfidptosis‐related genes

**FIGURE 12:**
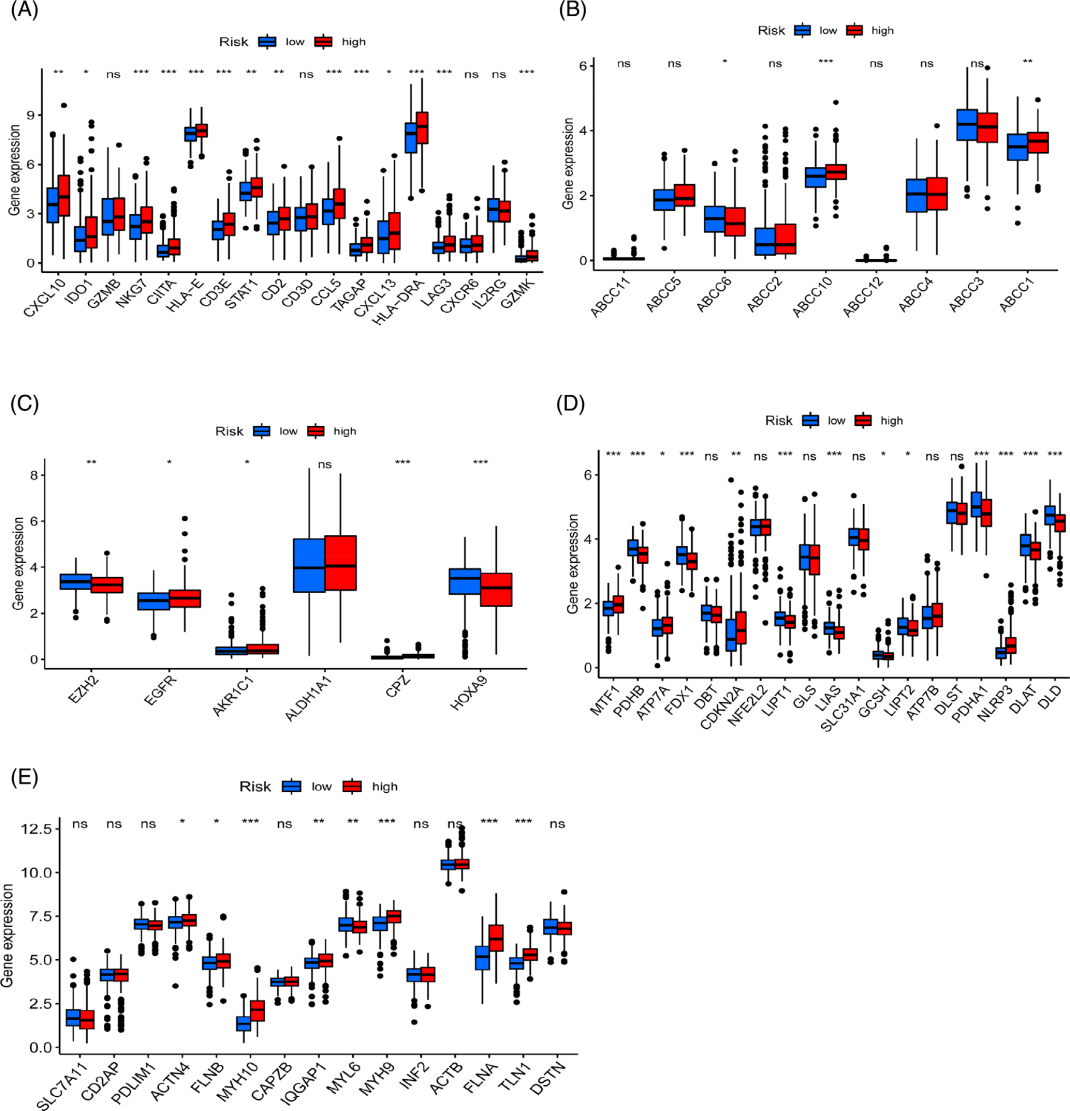
Several genes are expressed differently. (A–E) Differential expression analysis of TIS‐, multidrug resistance‐, chemoradiotherapy‐, cuproptosis‐, and disulfidptosis‐related genes. ***p < .001; **p < .01; *p < .05; ns, non significant.

The tumor inflammation index‐related genes are 18 based on IFN‐γA, a gene set composed of signaling pathways, T cell markers, and NK cell activity factors. These 18 genes are highly correlated with the clinical response of ICIs, and TIS can more effectively predict the clinical immunotherapy efficacy of patients compared to PD‐L1 protein expression. To determine whether high‐risk patients might benefit from therapeutic immunotherapy, we analyzed gene expression levels linked with TIS in high‐ and low‐risk groups and discovered a substantial difference (*p* < .05, Figure 12C). This finding is supported by the observation of a positive connection between the expression of genes associated with TIS and risk scores (Figure S6C). We also looked for links between the risk score and genes known to contribute to resistance to chemo, radiation, and combination drug regimens. Expression levels of ABCC10 and ABCC1 in many drug resistance‐related genes were considerably greater in the high‐risk group compared to the low‐risk group, whereas expression levels of ABCC6 were significantly higher in the low‐risk group compared to the high‐risk group (*p* < .05, Figure 12B). The expression levels of ABCC6 and ABCC3 in these genes are negatively connected to risk scores (Figure S6B). Increased expression of radiation and chemotherapy‐related genes such as EZH2 and HOXA9 was seen in the low‐risk group (*p* < .05, Figure 12D), and there was a negative link with the risk score and these genes (Figure S6D). EGFR, AKR1C1, and CPZ were more significantly expressed in the high‐risk group and positively link with risk scores. Cuproptosis and disulfidptosis are two newly discovered cell death modes. Among the 19 cuproptosis‐related genes, 13 showed differences in expression levels between the two groups. The high‐risk group had elevated levels of expression for MTF1, CDKN2A, and NLRP3, whereas the low‐risk group had elevated expression for the other 10 genes (*p* < .05, Figure 12A). Similar to the findings of level differences, evidence of a link between cuproptosis expression and risk score holds (Figure S6A). When comparing the two groups, we found that ACTN4, FLNB, MYH10, IQGAP1, MYH9, FLNA, and TLN1 expression levels were all greater in the former (*p* < .05, Figure 12E), whereas the low‐risk group had elevated levels of just MYL6. The association between risk score and disulfidptosis‐related gene expression supports the results of our differential analysis (Figure S6E).

### 3.11 Drug sensitivity analysis

To find suitable targeted drugs and chemotherapy drugs for treating CRC patients with an MRG risk score, risk score, and half‐inhibitory concentration were analyzed together. Cytarabine and GW.441756 exhibited lower IC50 values in the low‐risk category (*p* < .001, Figure S7A,B). High‐risk CRC patients responded better to treatments such AZD.2281, bexarotene, TW.37, and dasatinib (*p* < .001, Figure S7C–F). The sensitivity to drugs indicates the effectiveness that can be achieved in radiotherapy and chemotherapy, indicating that we may have found suitable treatment drugs for MRG risk scoring.

### 3.12 Expression level and immunohistochemistry validation of model genes

Five model genes were selected and their expression differences between normal colon tissue and CRC tumor tissue were retrieved using the TCGA cohort. HIGD1A, EFHD1, and PPARGC1A were found to be overexpressed in normal tissue, whereas TRAP1 and P4HA1 were found to be overexpressed in CRC tumor tissue (*p* < .001, Figure S8A). Except for PPARGC1A, for which pathological and normal tissue samples could not be obtained, the above results were validated by the expression levels of four other genes between tumor and healthy tissues, as shown by the immunohistochemistry results acquired from the HPA database (Figure S8B,C).

### 3.13 Five MRGs in scRNA‐Seq

To investigate TME MRGs expression, we used the TISCH2 database's single cell dataset GSE166555 from CRC patients. It can be seen from the data that the GSE166555 dataset has 13 different kinds of cells (Figure S9A). TRAP1 and P4HA1 are highly expressed at the intracellular level, whereas PPARGC1A, EFHD1, and HIGD1A are only expressed by a minority of tumor cells (Figure S9B). TRAP1 and P4HA1 are mainly expressed on malignant cells, CD4+ conventional T cells, and fibroblasts. Epithelia cells mainly express a small amount of TRAP1, P4HA1, PPARGC1A, and HIGD1A. The expression of EFHD1 is mainly concentrated in myofibroblasts.

### 3.14 Pan‐cancer analysis of model genes

Pan‐cancer expression profiling revealed that all 5 MRGs were differently expressed across a wide range of tumor types. Among them, TRAP1 is highest expressed in COAD and READ, while P4HA1 is highest expressed in KIRC, GEM, and HNSC (Figure S10A). The expression of TRAP1 in pan‐cancer was shown to be substantially inversely linked with that of HIGD1A and EFHD1, and positively correlated with that of PPARGC1A, according to a correlation study of 5 MRGs (Figure S10B). P4HA1 and EFHD1 expression levels are positively linked. Then, we looked at the role that model genes have in pan‐cancer prognosis. While TRAP1 is protective in terms of prognosis in LGG and READ, it poses a danger in LAML (Figure S10C). HIGD1A is a protective factor in MESO. EFHD1 is a protective factor in KICH, KIRC, and LGG, while it is a risk factor in COAD. PPARGC1A is a favorable factor in ACC, COAD, KIRC, KIRP, and MESO, while it is an unfavorable factor in THCA, THYM, and UCEC. P4HA1 is a risk factor in various cancers, such as CESC, HNSC, KICH, KIRP, MESO, PAAD, SARC, THCA, and UVM. In summary, five MRGs play different roles in the prognosis of different cancers.

## 4 DISCUSSION

CRC is a cancer of the digestive tract that has consistently ranked high in the global rankings of cancer incidence rate and death. The survival rate for people with CRC has grown as a result of the constant development of new treatments in recent years.^64^, ^65^ Unfortunately, individuals with advanced and metastatic CRC still have a relatively low life expectancy because of problems such tumor heterogeneity. Therefore, finding effective biomarkers and constructing accurate prognostic models are particularly crucial. Mitochondria are crucially involved in metabolic reprogramming and cell death. The structure and function of mitochondria are affected by MRGs.^34^, ^66^ Although MRGs have been shown to be useful indicators for prognosis and immunotherapy in a variety of tumor types, not enough is known about their role in CRC yet.

We conducted this research to learn more about the link between MRG expression and clinical outcomes and immunotherapy for CRC. DEGs in the mitochondria were examined, and existing CRC samples were categorized. We found that after NMF clustering, the OS and PFS rates of CRC patients in Cluster 1 were higher than those in Cluster 2. But when we evaluated the two clusters' immunological scores on the basis of the amount of TME and immune cell infiltration, we observed no significant difference. Cluster2 had a considerably worse prognosis than Cluster1, likely because of its higher matrix score and estimate score. A higher matrix score indicated a greater propensity to tumor cell metastasis.^67^ Analysis of immunological subtypes for Clusters 1 and 2 found no statistically significant variations in subtype assignment. Cluster 2 had more T cells, endothelial cells, and fibroblasts, according to an examination of immune cell infiltration. Tumor parenchymal cells rely on interstitial cells like endothelial cells and fibroblasts for nourishment and growth, which in turn affects tumor parenchyma's ability to invade and metastasize.^68^, ^69^

To further assess the predictive usefulness of MRGs in CRC patients, we created a risk score‐based prognosis model for mitochondrial‐related characteristics. Groups of high and low risk were determined using a model on the basis of five MRGs (TRAP1, HIGD1A, PPARGC1A, EFHD1, and P4HA1). The low‐risk group demonstrated strong prognostic value in the Kaplan–Meier and ROC curves of OS and PFS rates. Patients' outcomes are correlated with TNM staging, and our prognostic model shows high prediction power for a variety of clinical and pathological characteristics. In subgroup analysis of clinical pathological features, our prognostic model also shows better application value, especially in stages T, N, and M. Independent prognostic research confirms that the MRG‐based risk score is a reliable predictor of survival in CRC patients. Moreover, a nomogram with greater accuracy and consistency in predicting CRC patients' survival was constructed.

TNF‐receptor‐associated protein‐1 (TRAP1) is a member of the Hsp90 family exclusive to mitochondria and a major regulator of metabolic flux. It can promote the occurrence and development of many cancers by upregulating its expression, supporting glycolysis, and counteracting cell death.^70^, ^71^ Interestingly, the upregulation of TRAP1 expression has different effects in different cancers, exhibiting a dual effect of carcinogenesis^72^, ^73^ or cancer inhibition.^74^ Clinical experiments have shown that overexpression of TRAP1 is connected to a poor prognosis in CRC patients.^75^ Apart from that, TRAP1 can regulate glycolysis metabolism and help reverse the resistance of CRC patients to EGFR inhibitors.^76^ Our immunohistochemical results confirm the conclusion that TRAP1 expression levels are upregulated in CRC patients compared to normal colon tissue. Hypoxia‐inducible gene domain family member 1A (HIGD1A) is a HIF‐1α The regulated mitochondrial proteins can regulate oxygen consumption and reactive oxygen species production under hypoxic conditions, thereby activating the dormancy mechanism of tumor cells and enabling their survival.^77^, ^78^ Related studies have shown that silencing the expression of HIGD1A can inhibit the malignant development of tumors.^79^ Nonetheless, studies on a variety of colon adenocarcinoma cell lines^80^ show that suppressing HIGD1A expression may stimulate tumor cell motility and invasion, promote colony formation, and increase cell proliferation. Peroxisome proliferator activated receiver gamma coactivator 1 alpha (PPARGC1A) is a key transcription coactivator that can regulate oxidative phosphorylation to promote tumor cell metastasis.^81^ Upregulation of PPARGC1A expression can promote the metastasis of lung cancer.^82^ However, the link between increased PPARGC1A expression and a favorable prognosis in clear cell renal cell carcinoma remains contentious.^83^ The mutation of PPARGC1A has been shown to contribute to CRC susceptibility^84^ and can serve as a negative biomarker for CRC.^85^, ^86^ EF hand domain family member D1 (EFHD1) is a calcium‐binding protein located in the inner mitochondrial membrane.^87^ The combined detection of EFHD1 and PPP1R3C methylation in plasma DNA tests of CRC patients can show high sensitivity and specificity,^88^ this might aid in the early detection of CRC patients. Prolyl 4‐hydroxylase bundle 1 (P4HA1) participates in extracellular matrix remodeling^89^, ^90^ by regulating HIF‐1α. Additionally, it may stimulate tumor invasion, metastasis, and the EMT transition.^91^, ^92^ The P4HA1/HIF1 has been identified as a modulator of glycolysis and carcinogenic activity in pancreatic cancer.^93^ Previous research has shown that P4HA1 is a predictive marker for both oral squamous cell carcinoma and high‐grade glioma.^94^, ^95^ Important to note, we assessed the predictive performance of the prognostic models composed of these five genes with other models, and the results showed that our prognostic model has better predictive value and better clinical practicality. Meanwhile, the prognostic model used as a control comparison is based on ferroptosis‐related genes, which have been widely studied in current research and are gradually recognized as reliable biomarkers. Therefore, our prognostic model has more reliable results than other models, which may mean that these five MRGs can also serve as biomarkers guiding the clinical prognosis of CRC patients.

In order to learn more about the results of MRG on immunotherapy, we next performed a deep investigation on the connection between MRG and the tumor immune microenvironment (TIME). The MRG risk score was first subjected to a bioconcentration study, a GO enrichment analysis showed that the matrix has strong ties to the high‐risk cohort. According to the findings of the KEGG enrichment pathway analysis, we know that CAMs, ECM receptor interaction, cycline receptor interaction, various cancer‐related pathways, and classical carcinogenic pathways contribute substantially to cancer occurrence in the high‐risk population. We use a variety of TIME‐related algorithms to comprehensively assess the immune landscape of the MRG risk score. The high‐risk group scored higher on all three measures (matrix, immunology, and ESTIMATE), as well as poorer tumor purity, according to the ESTIMATE‐based TME data. Tumor stromal cells, which are abundant in tumor tissue and thought to help with tumor development, illness progression, and medication resistance, are reflected in the matrix score.^96^ We used bioaccumulation analysis results to examine the intricate relationship between tumor cells, stromal cells, and extracellular matrix in the TME, which acts as a biological barrier around tumor tissue, prevents immune cells from penetrating tumor sites, and makes the tumor resistant to radiation, chemotherapy, and immunotherapy.^97^, ^98^ These factors may contribute to a poor outcome for patients in the high‐risk category. The prognosis of individuals with CRC is strongly influenced by the variety of immune cells that have infiltrated the tumor. Using the MCPcouter algorithm, we discovered that there is a positive correlation between the presence of many immune cells and risk scores. This finding suggests that the infiltration content of immune cells is higher in the high‐risk category. At this point, our supplementary algorithmic analysis showed that M0 macrophages and resting NK cells constituted the bulk of the immune cell types in the high‐risk group, both of which showed a greater infiltration abundance. Macrophages in the TIME are usually called TAM, the M2 macrophage activation subtype, enhances tumor cell EMT and angiogenesis, therefore facilitating CRC liver metastasis.^99^, ^100^, ^101^, ^102^ M0 macrophages can be induced by anti‐inflammatory factors^103^ to polarize into M2 macrophages with immunosuppressive effects,^104^ thereby promoting the CRC development. Extensive research has demonstrated that NK cells have an anti‐tumor role as congenital effectors in gastrointestinal cancers, one of their many TME functions.^105^ However, in CRC patients, tumor‐related NK cells have been found to secrete angiogenic factors through the STAT3 and STAT5 pathways to stimulate tumor blood vessel formation and invasion.^106^, ^107^

In B‐ and T‐cell acute lymphoblastic leukemia (B/T‐ALL), studies have linked significant infiltration of NK cells to a poor prognosis. Based on our research, it can be inferred that a high infiltration of tumor‐related NK cells in CRC patients may indicate a poor prognosis. In addition, our combined study of immune cell and survival data in CRC patients revealed that an abundance of T cells follicular helper and mast cell activation is associated with a better prognosis than a plethora of naive B cells and M0 macrophages infiltration. Immune function scores and levels of immune checkpoint genes were compared between the two groups using ssGSEA, and the high risk group was found to have considerably higher scores and levels for the majority of the immunological checkpoint genes. Among the most widely‐studied immune checkpoint genes, CD274, CTLA4, and PDCD1 all show positive correlations with risk assessments. Inhibition of immune checkpoint expression promotes immunological responses against tumors and has a suppressive effect. ICI therapy is currently a mature and widely used means of immunotherapy. Important immunosuppressive molecules such as PD‐1 and CTLA‐4 enhance tumor incidence and growth by aiding in the evasion of immune surveillance.^108^, ^109^ In CRC patients, the up‐regulated expression of PD‐1 may indicate poor prognosis,^110^ and CTLA‐4 can inhibit the proliferation of T‐effector cells, thereby inhibiting tumor immunity.^111^ Together with previous findings on the number of immune cells in different organs, this data suggests that the compensatory phenomenon of local immune activity being lowered by checkpoints may explain the substantial infiltration of immune cells in the high‐risk group. A high number of T cells and other immune cells congregate in the tumor's periphery, yet they are unable to enter the cancer nest. With the increase of compensatory immunosuppressive cells, the absolute increase of certain types of immune cells is limited, ultimately resulted with a bad outlook in CRC patients. The TIDE and IPS scores were then examined to determine the efficacy of immunotherapy. The evaluation using the TIDE score represents tumor evasion of immunity, while a higher TIDE score in the high‐risk group implies a higher likelihood of immune surveillance evasion, suggesting that ICI therapy is less likely to be successful for high‐risk individuals. Results from the IPS score suggest that immunotherapy with PD‐1 or CTLA4 inhibitors alone is most effective for patients in the low‐risk category. These results provide more evidence that the MRG risk score corresponds with immunotherapy effectiveness.

We performed further research on the association between the risk score and the MSI, TMB, and CSC indices to more thoroughly examine and forecast the response of the MRG risk score to immunotherapy effectiveness. When comparing the proportion of patients with MSS and MSI‐H type CRC, more MSI‐H cases were found in the high‐risk group than in the low‐risk group. Previous research has used MSI status to categorize CRC patients. MSI‐H type CRC belongs to a “hot tumor” that is sensitive to immunotherapy,^112^ while MSS type CRC belongs to a “cold tumor” that is not sensitive to immunotherapy. Patients who score poorly on both the TIDE and IPS have a greater chance of benefiting from immunotherapy when MSI results are taken into account. However, patients who score highly on both the TIDE and IPS, as well as those with a high MSI‐H proportion, can become the “dominant population” for immunotherapy in the high‐risk group. Higher degrees of cell differentiation and less stem cell features are seen in CRC cells with lower MRG risk scores, the MRG risk score is inversely related to the CSC index. A strong correlation has been identified in CRC between MSI and TMB at high levels. The high TMB reflects a higher number of new antigens released or presented by tumors, which can stimulate the response of immune cells (especially T cells), thereby enhancing the therapeutic effect.^113^ The correlational findings of this research showed that the presence of TMB increased as the MRG risk score increased. A better prognosis may be expected with immunotherapy in individuals who have both high TMB and low risk scores. The limits of biomarkers, such as TMB, are increasingly being brought to light, despite growing evidence that this is necessary due to the intricacy of the immunological process in the TME,^114^ so it is more necessary to cooperate with other predictors to better apply them to clinical practice. The data from previous investigations indicate that mitochondria are engaged in cell metabolism, proliferation, and programmed cell death, affecting tumor cell death, drug resistance, and the immune response. TIS is a new tumor immune biomarker, and multiple drug resistance is the main barrier to the success of CRC chemotherapy. Cuproptosis and disulfidptosis are newly discovered cell death modes that may serve as potential targets for inhibiting cancer occurrence. By examining this link between the MRG risk score and the aforementioned associated genes, we give further suggestions for our prognostic model in clinical therapy. Based on the risk score, we predicted drug sensitivity and identified six potential drug targets related to the MRG risk score. In addition, scRNA‐seq results can determine the expression distribution of MRG in tumor cells, which is helpful for future gene expression localization and mechanism analysis. Finally, we performed a pan‐cancer examination of MRG to improve its predictive ability and potential applications in cancer types other than CRC. Our research is currently one of the most comprehensive and in‐depth articles on bioinformatics mining and analysis of mitochondrial features related to CRC. However, this article still has certain limitations, such as the lack of sufficient in vitro and in vivo experiments and real clinical data to verify the analysis results. However, our research findings still have certain guiding significance. With the continuous in‐depth analysis and exploration of mitochondrial function, its role in immune response has been continuously explored, especially in mitochondrial metabolism. It has been proven that improving mitochondrial metabolism can increase the response to anti‐PD‐1 antibodies, which is a promising method for treating different malignant tumors.^115^, ^116^ Some ions may interfere with mitochondrial metabolism and immune regulation by affecting oxidative stress and antioxidant defense barriers, such as magnesium ions.^117^ These results can play an important guiding role in personalized tumor treatment in our clinical practice.

## 5 CONCLUSION

In summary, our research indicates that MRG is a promising biomarker that is related to immunity and prognosis. By constructing and validating a prognostic model and comprehensively analyzing the response of the MRG risk score to immunotherapy and the correlation of multiple biomarkers, it can help guide the MRG risk score to become an effective tool for clinical application in CRC. The results of our studies aid in identifying the immunological profile of CRC patients and making more accurate therapy decisions.

## AUTHOR CONTRIBUTIONS

**Yun‐hui Xie:** Conceptualization (equal); data curation (supporting); formal analysis (equal); funding acquisition (lead); investigation (equal); methodology (equal); project administration (equal); resources (equal); software (equal); supervision (supporting); validation (supporting); visualization (supporting); writing – original draft (equal); writing – review and editing (equal). **Hui‐zhong Jiang:** Conceptualization (equal); data curation (lead); formal analysis (equal); funding acquisition (supporting); investigation (equal); methodology (equal); project administration (equal); resources (equal); software (equal); supervision (lead); validation (lead); visualization (lead); writing – original draft (equal); writing – review and editing (equal).

## FUNDING INFORMATION

This work was partially supported by the National Natural Science Foundation of China (No. 82170887); Chengdu High‐level Key Clinical Specialty Construction Project.

## CONFLICT OF INTEREST STATEMENT

The authors have stated explicitly that there are no conflicts of interest in connection with this article.

## ETHICS STATEMENT

All information used in this study came from a publicly available database or a published source that was properly cited. It is therefore not subject to ethical approval and informed consent is not required.

## Supporting information


**Data S1.** Supplementary material.
**Figure S1.** Verification of the MRG risk score‐based prognostic model.
**Figure S2.** Prognostic effect test of the prognostic models.
**Figure S3.** MRG risk score correlation with prognostic model.
**Figure S4.** Immune cell subtype association studies and prognostic value.
**Figure S5.** TMB correlation analysis and prognostic value.
**Figure S6.** MRG‐based correlation analysis with multiple genes.
**Figure S7.** Validation of prognostic model genes from single‐cell datasets.
**Figure S8.** Gene expression levels predicted by the MRG risk score were validated.
**Figure S9.** Validation of prognostic model genes from single‐cell datasets.
**Figure S10.** Pan‐cancer MRG risk score‐based predictive model gene expression and clinical utility.
**Table S1.** Basic information on clinical data of TCGA.

## Data Availability

The datasets used in this research are publicly available via several databases. Article/Supplementary Materials contains information about repositories and accession numbers.
