# Supplementary material for: The exploration of mitochondrial‐related features helps to reveal the prognosis and immunotherapy methods of colorectal cancer
Source: Cancer Rep (Hoboken). 2023 Oct 30;7(1):e1914. doi: 10.1002/cnr2.1914 (PMC10809275; doi:10.1002/cnr2.1914)
Supplement: Supplementary file 1 — Data S1. Supplementary material. Figure S1. Verification of the MRG risk score‐based prognostic model. Figure S2. Prognostic effect test of the prognostic models. Figure S3. MRG risk score correlation with prognostic model. Figure S4. Immune cell subtype association studies and prognostic value. Figure S5. TMB correlation analysis and prognostic value. Figure S6. MRG‐based correlation analysis with multiple genes. Figure S7. Validation of prognostic model genes from single‐cell datasets. Figure S8. Gene expression levels predicted by the MRG risk score were validated. Figure S9. Validation of prognostic model genes from single‐cell datasets. Figure S10. Pan‐cancer MRG risk score‐based predictive model gene expression and clinical utility. Table S1. Basic information on clinical data of TCGA. [file CNR2-7-e1914-s001.docx]

Supplementary Material

# Supplementary Figures


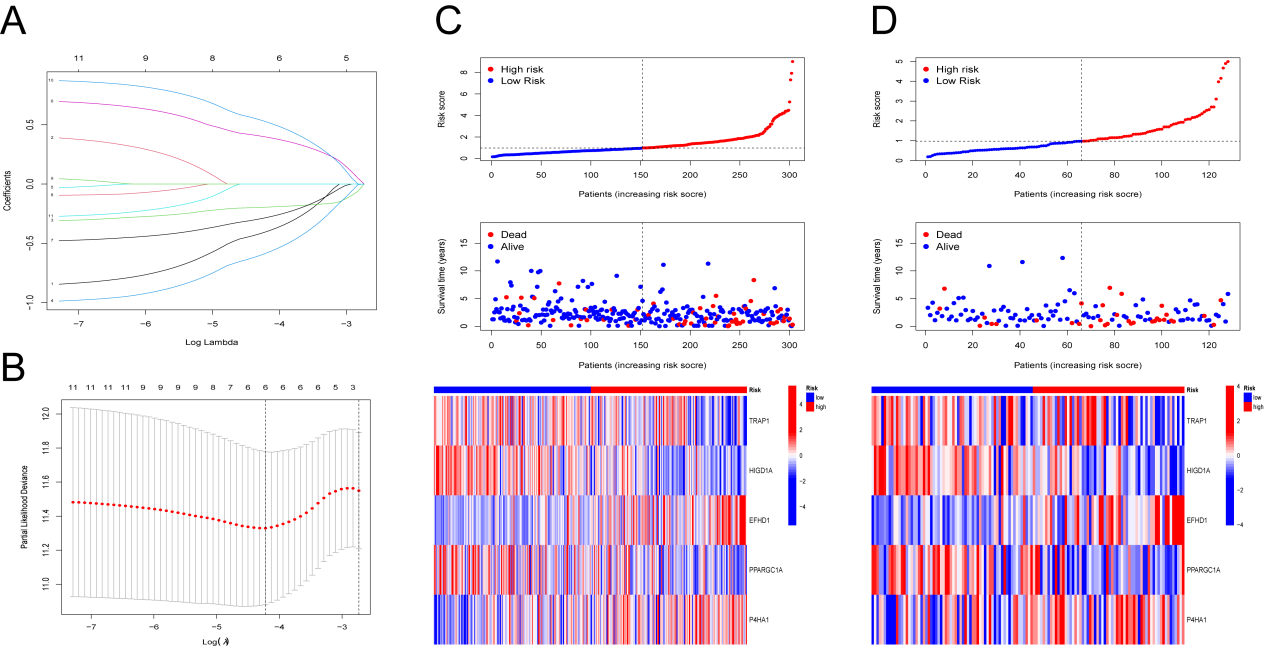


**Supplementary Figure S1.** Verification of the MRG risk score-based prognostic model. (A, B) For genes having prognostic traits, the Lasso technique was employed to generate coefficients; (C, D) Gene expression profiles linked to the MRG risk scores, their distribution, and the outcomes of the training and testing sets.


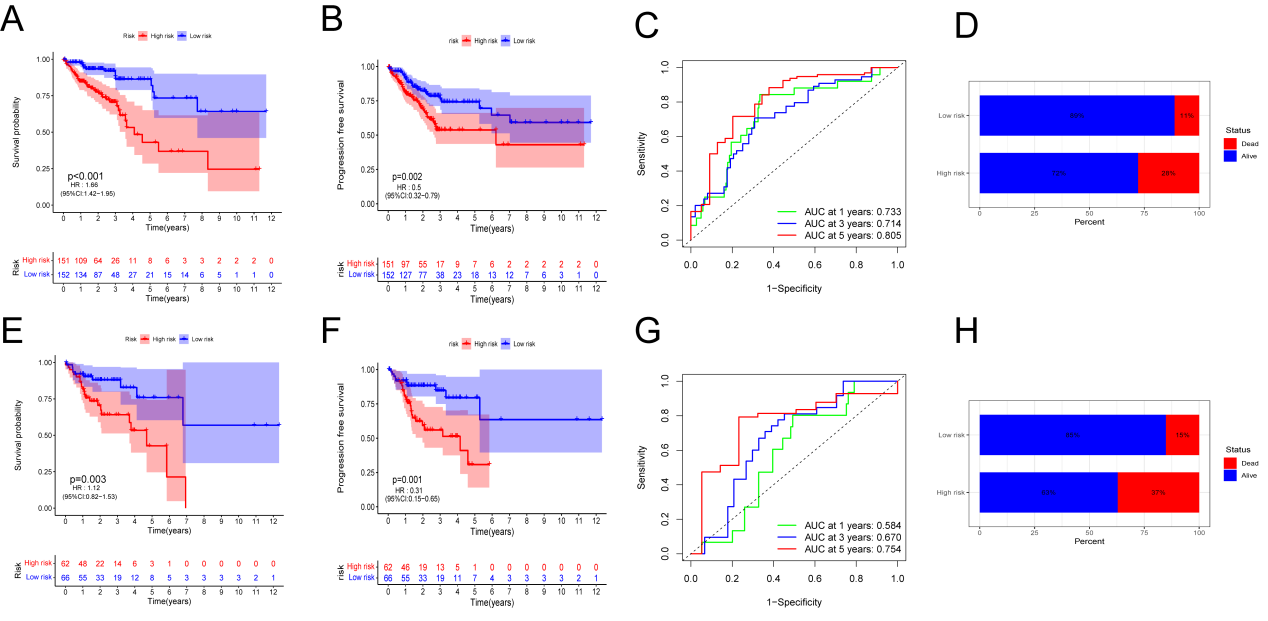


**Supplementary Figure S2.** Prognostic effect test of the prognostic models. (A, B, C, D) In the training set, results of the analysis of OS rate, PFS rate, ROC curves of survival prognosis, and survival ratio of patients ; (E, F, G, H) In the test set, results of the analysis of OS rate, PFS rate, ROC curves of survival prognosis, and survival ratio of patients.


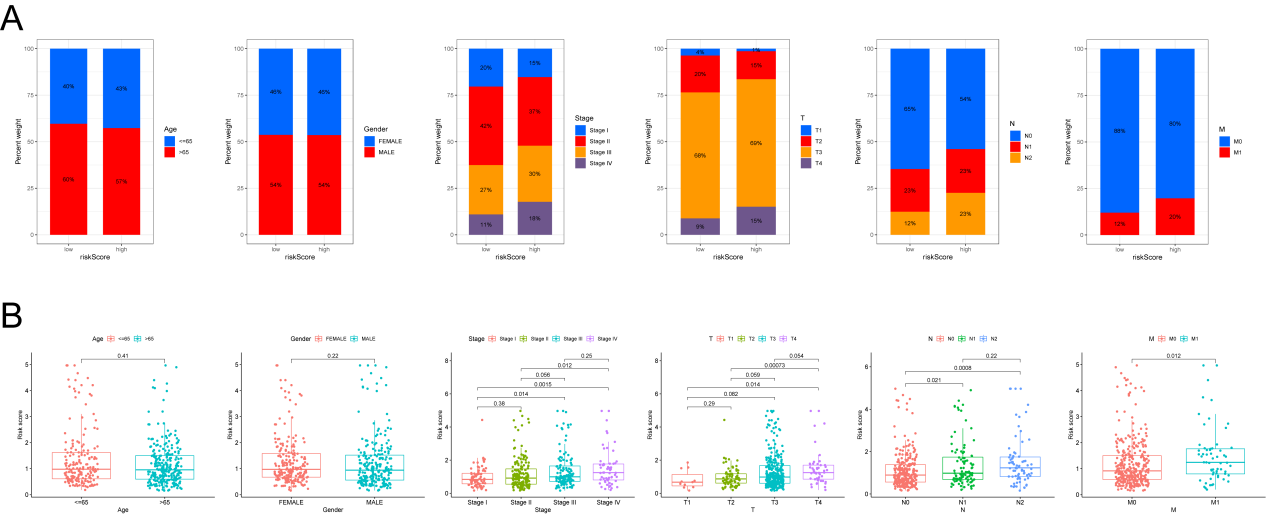


**Supplementary Figure S3.** MRG risk score correlation with prognostic model. (A) The ratio of low- and high- risk groups to clinicopathological features; (B) Clinical and pathological characteristics: correlation with MRG risk score.


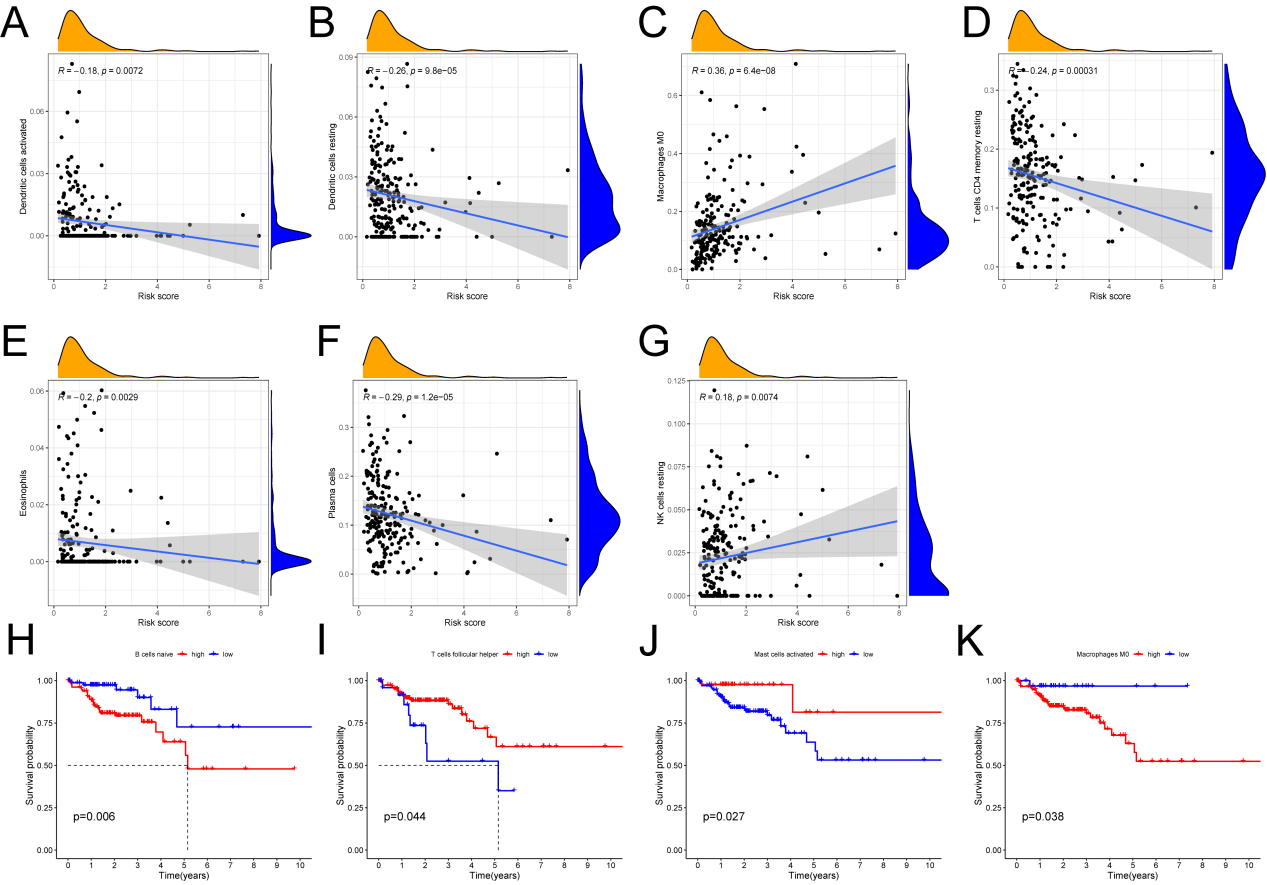


**Supplementary Figure S4.** Immune cell subtype association studies and prognostic value. (A-G) Results of the link between MRG risk score and immune cell subtypes; (H-K) Prognostic impact of immune cell infiltration levels.


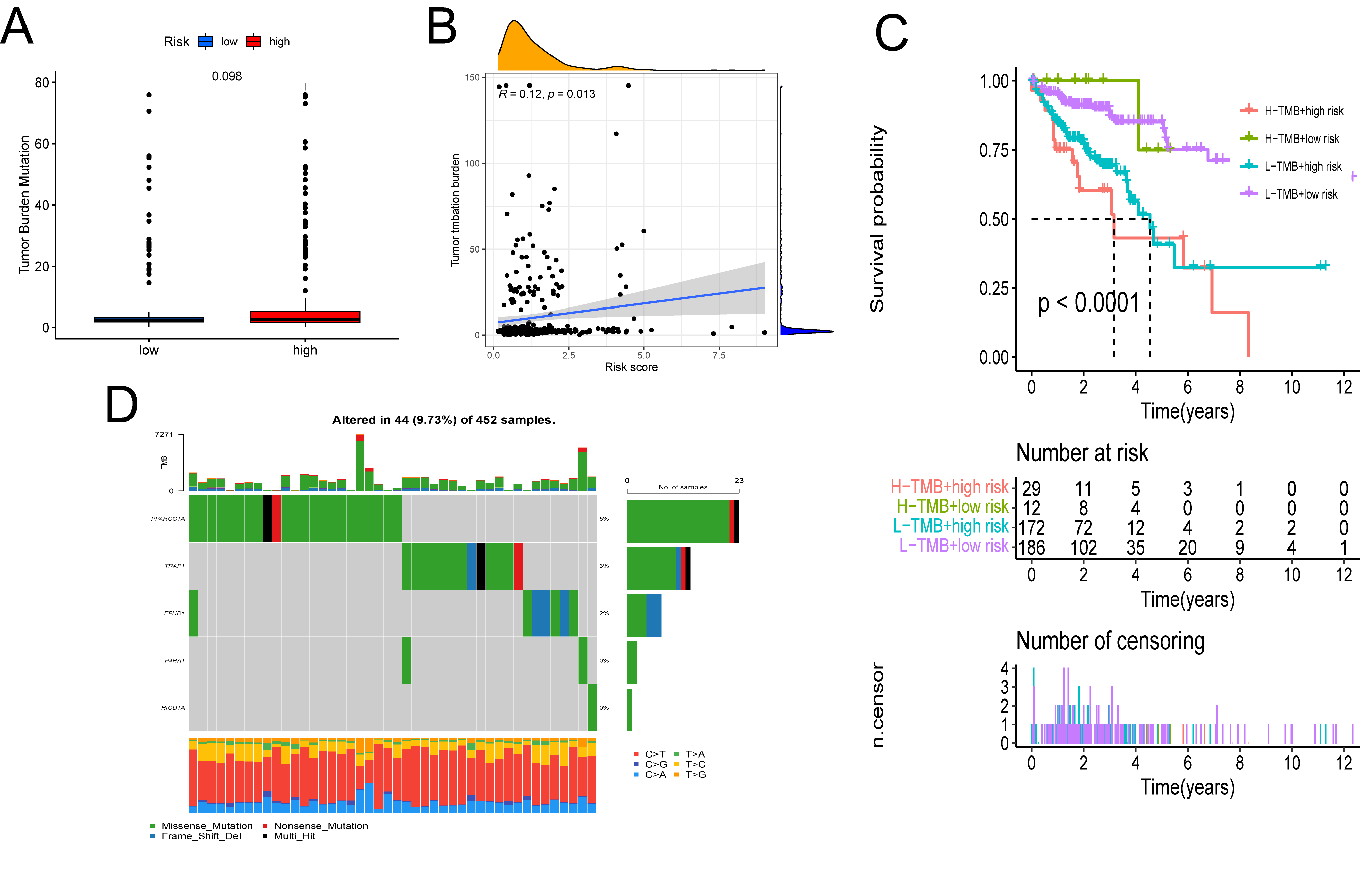


**Supplementary Figure S5.** TMB correlation analysis and prognostic value. (A) Analyzing the TMB variation between low and high risk groups; (B) Comparing the MRG risk score with the TMB using Spearman's correlation; (C) Contrast of the MRG prognostic score with the TMB；(D) Oncoplots showing the mutation results of the prognostic model genes.


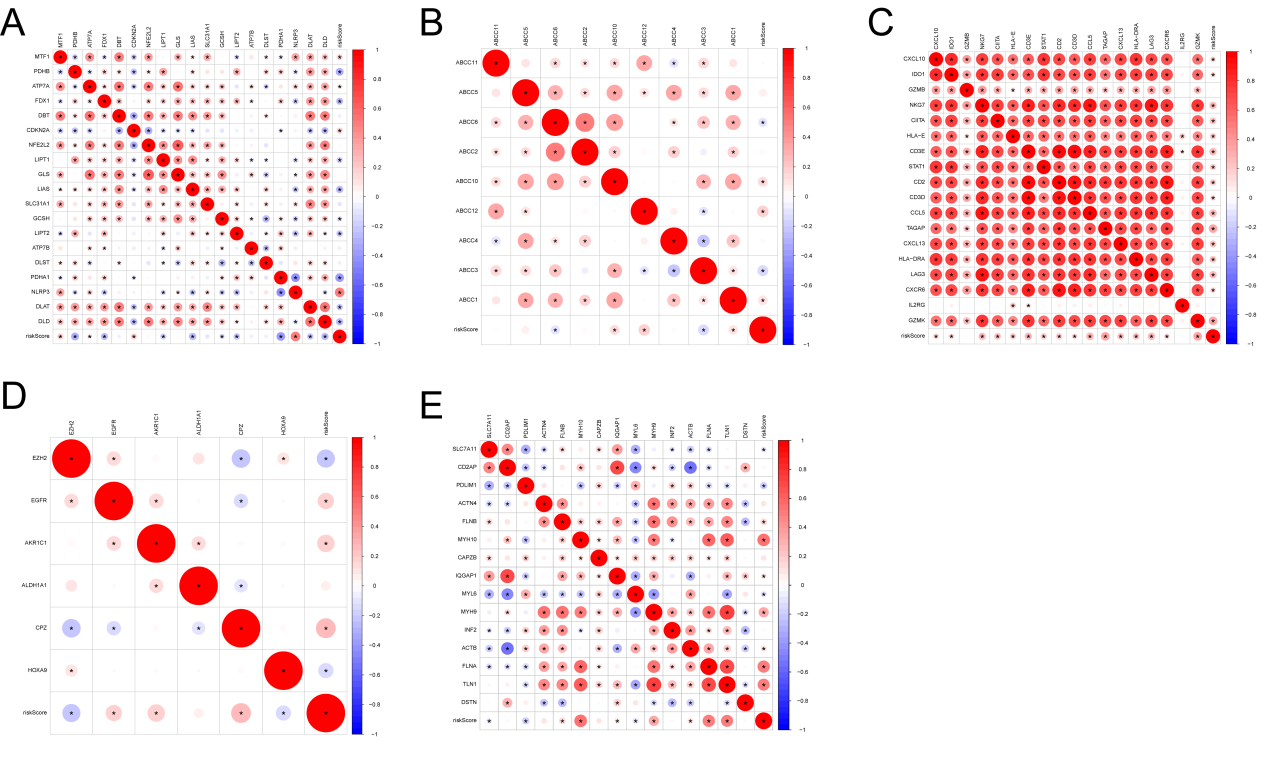


**Supplementary Figure S6.** MRG-based correlation analysis with multiple genes. (A, B, C, D, E) Results of correlation analysis between the two groups and genes related to cuproptosis-, multidrug resistance-, TIS-, chemoradiotherapy-, and disulfidptosis-related genes.


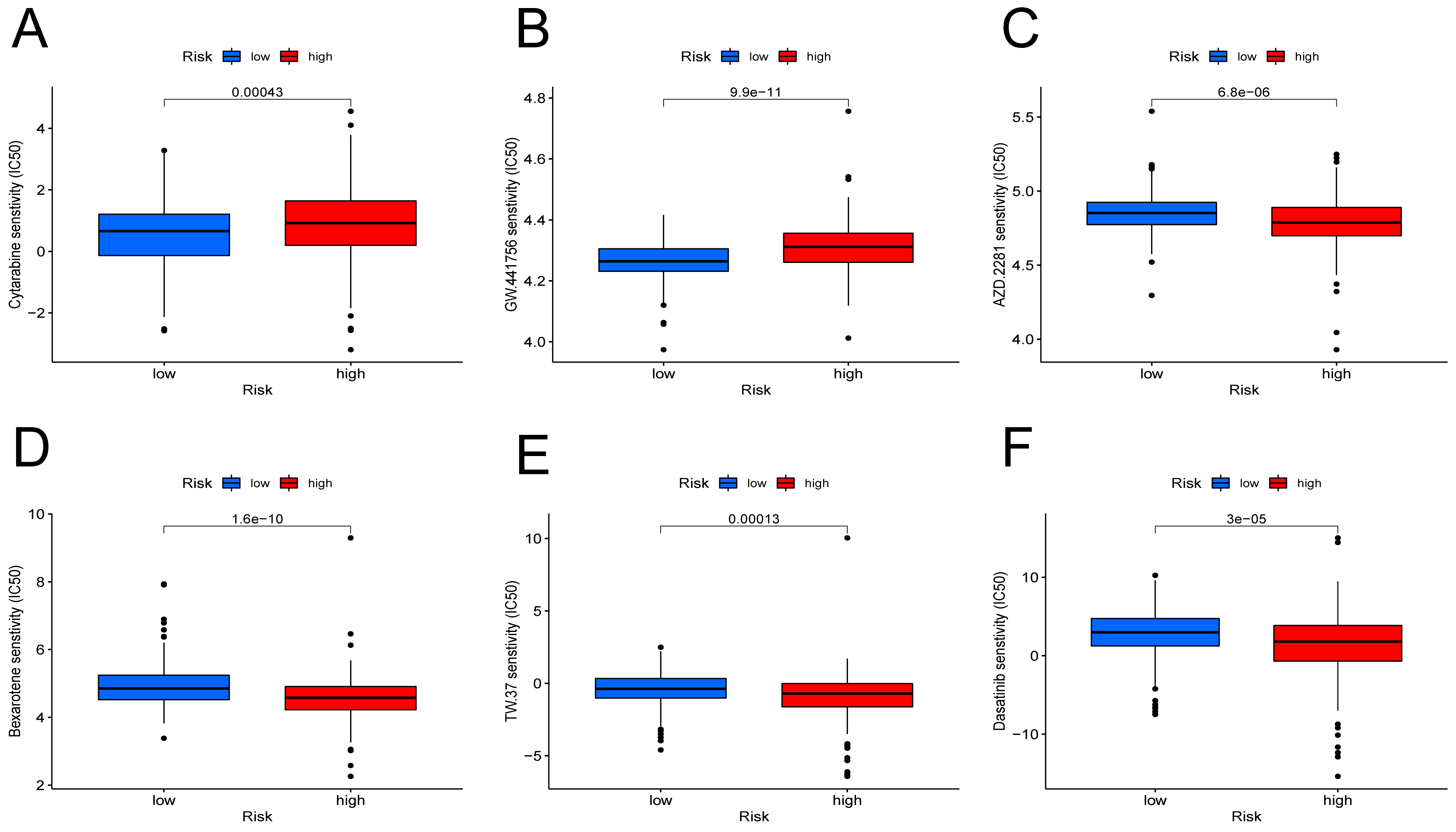


**Supplementary Figure S7.** Riskscores and drug sensitivity. (A) Cytarabine; (B) GW.441756; (C) AZD.2281; (D) Bexarotene; (E) TW.37; (F) Dasatinib.


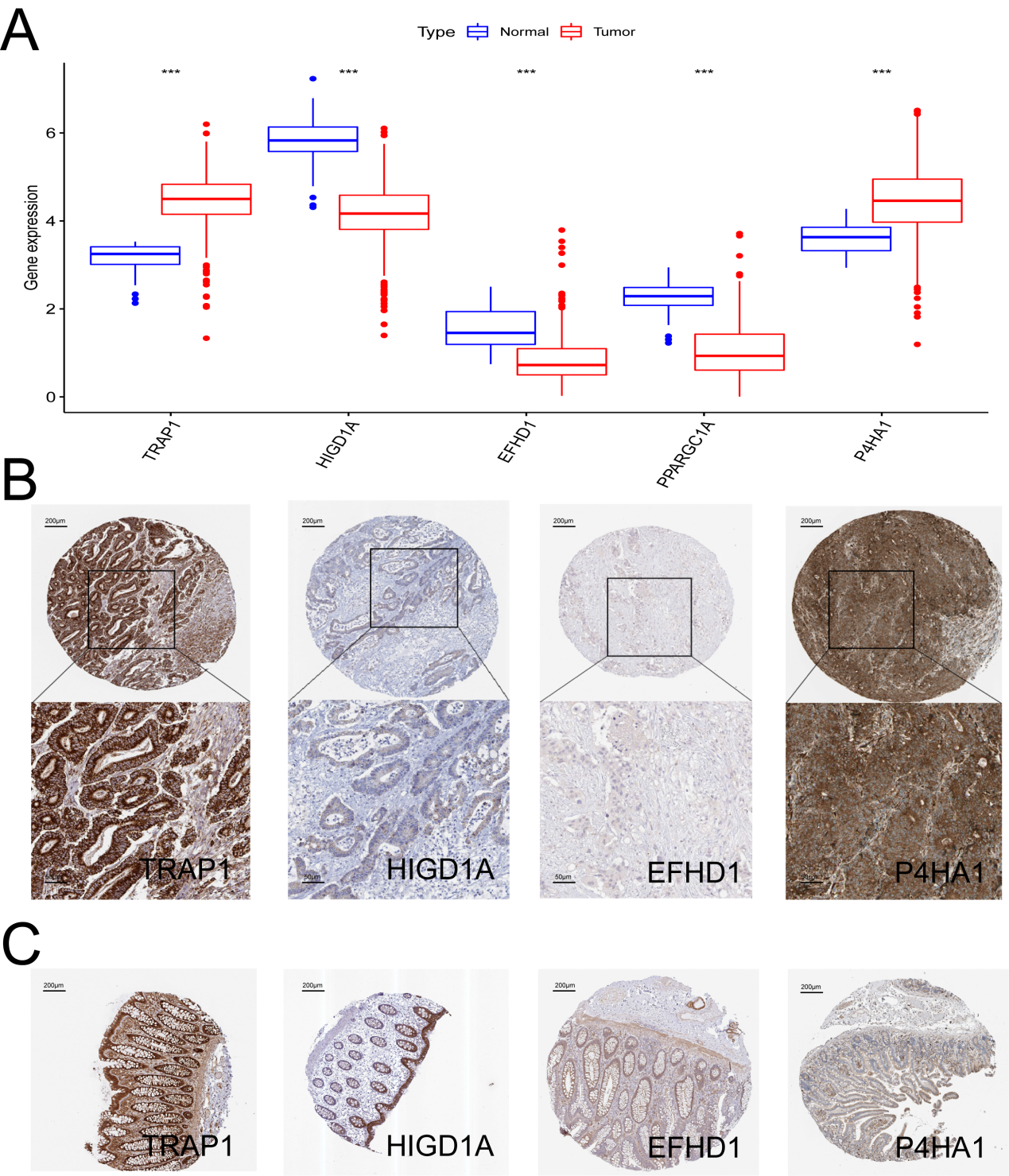


**Supplementary Figure S8.** Gene expression levels predicted by the MRG risk score were validated. (A) Gene expression profiling of the TCGA cohort: a comparison of normal and malignant tissue; (B) Immunohistochemical expression results of TRAP1, HIGD1A, EFHD1, and P4HA1 in CRC tumor tissues; (C) Immunohistochemical expression results of TRAP1, HIGD1A, EFHD1, and P4HA1 in colon normal tissues. ***, p < 0.001.


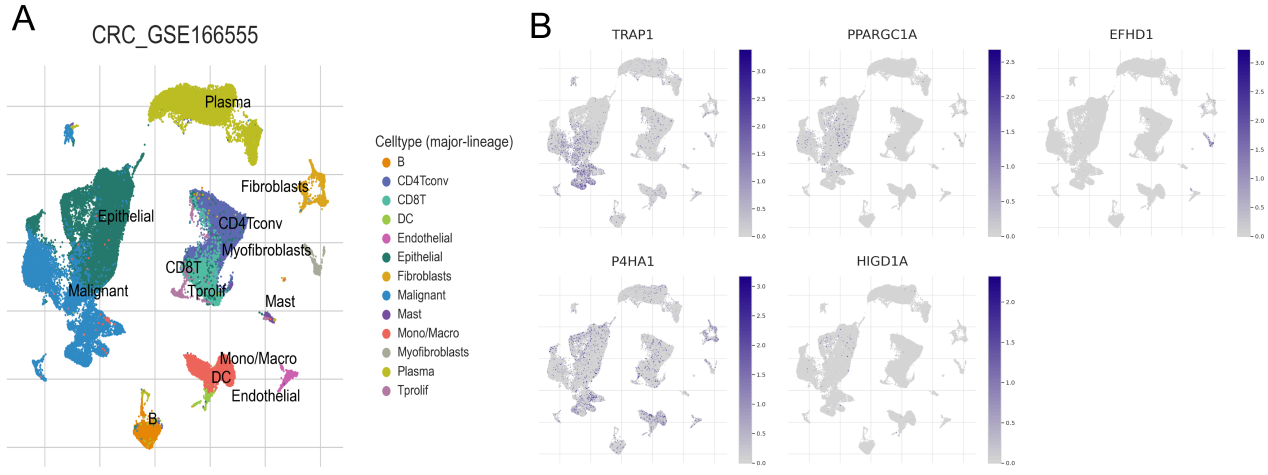


**Supplementary Figure S9.** Validation of prognostic model genes from single-cell datasets. (A) 13 cell types were divided in the tumor tissues of CRC patients; (B) Results of the five prognostic genes in cells.


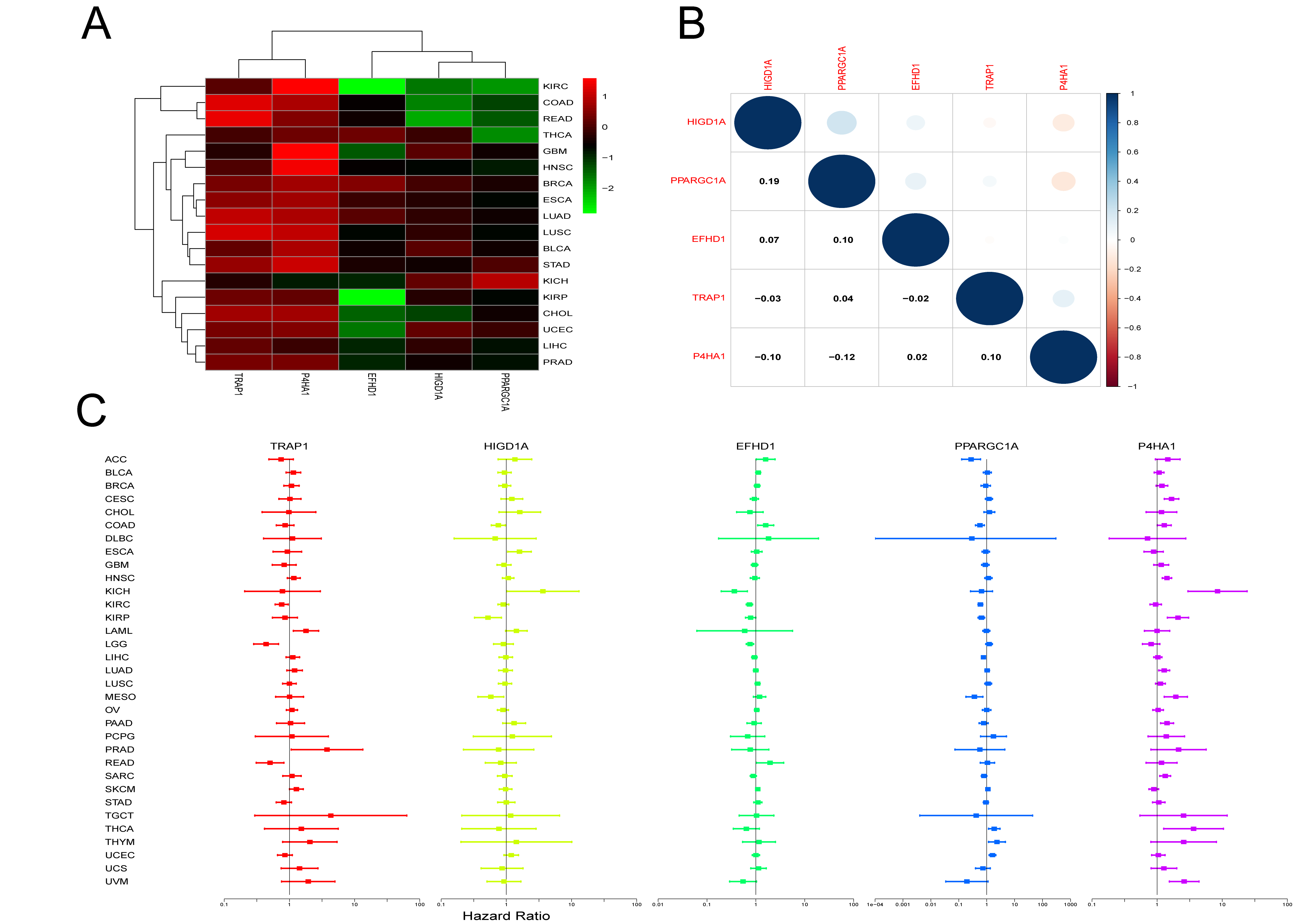


**Supplementary Figure S10.** Pan-cancer MRG risk score-based predictive model gene expression and clinical utility. (A) Gene expression profiling for pan-cancer prognosis; (B) Results of the correlation analysis of the prognostic genes; (C) 5 genes were investigated for their predictive usefulness in pan-cancer using Cox analysis.

# Supplementary Tables

# Supplementary Table S1. Basic information on clinical data of TCGA.

| Covariates | Type | Total | Test | Train | Pvalue |
| --- | --- | --- | --- | --- | --- |
| Age | <=65 | 179(41.53%) | 53(41.41%) | 126(41.58%) | 1 |
| Age | >65 | 252(58.47%) | 75(58.59%) | 177(58.42%) |  |
| Gender | FEMALE | 200(46.4%) | 58(45.31%) | 142(46.86%) | 0.8497 |
| Gender | MALE | 231(53.6%) | 70(54.69%) | 161(53.14%) |  |
| Stage | Stage I | 75(17.4%) | 23(17.97%) | 52(17.16%) | 0.3071 |
| Stage | Stage II | 166(38.52%) | 50(39.06%) | 116(38.28%) |  |
| Stage | Stage III | 119(27.61%) | 28(21.88%) | 91(30.03%) |  |
| Stage | Stage IV | 60(13.92%) | 22(17.19%) | 38(12.54%) |  |
| Stage | unknow | 11(2.55%) | 5(3.91%) | 6(1.98%) |  |
| T | T1 | 11(2.55%) | 2(1.56%) | 9(2.97%) | 0.7262 |
| T | T2 | 75(17.4%) | 25(19.53%) | 50(16.5%) |  |
| T | T3 | 293(67.98%) | 86(67.19%) | 207(68.32%) |  |
| T | T4 | 51(11.83%) | 14(10.94%) | 37(12.21%) |  |
| T | unknow | 1(0.23%) | 1(0.78%) | 0(0%) |  |
| N | N0 | 256(59.4%) | 78(60.94%) | 178(58.75%) | 0.6576 |
| N | N1 | 100(23.2%) | 31(24.22%) | 69(22.77%) |  |
| N | N2 | 75(17.4%) | 19(14.84%) | 56(18.48%) |  |
| M | M0 | 320(74.25%) | 88(68.75%) | 232(76.57%) | 0.2 |
| M | M1 | 60(13.92%) | 22(17.19%) | 38(12.54%) |  |
| M | unknow | 51(11.83%) | 18(14.06%) | 33(10.89%) |  |
